# A Chromosome Segment Substitution Library of Weedy Rice for Genetic Dissection of Complex Agronomic and Domestication Traits

**DOI:** 10.1371/journal.pone.0130650

**Published:** 2015-06-18

**Authors:** Prasanta K. Subudhi, Teresa De Leon, Pradeep K. Singh, Arnold Parco, Marc A. Cohn, Takuji Sasaki

**Affiliations:** 1 School of Plant, Environmental, and Soil Sciences, Louisiana State University Agricultural Center, Baton Rouge, Louisiana, United States of America; 2 Department of Plant Pathology and Crop Physiology, Louisiana State University Agricultural Center, Baton Rouge, Louisiana, United States of America; 3 Tokyo University of Agriculture, Tokyo, Japan; International Rice Research Institute, PHILIPPINES

## Abstract

Chromosome segment substitution lines (CSSLs) are a powerful alternative for locating quantitative trait loci (QTL), analyzing gene interactions, and providing starting materials for map-based cloning projects. We report the development and characterization of a CSSL library of a U.S. weedy rice accession ‘PSRR-1’ with genome-wide coverage in an adapted rice cultivar ‘Bengal’ background. The majority of the CSSLs carried a single defined weedy rice segment with an average introgression segment of 2.8 % of the donor genome. QTL mapping results for several agronomic and domestication traits from the CSSL population were compared with those obtained from two recombinant inbred line (RIL) populations involving the same weedy rice accession. There was congruence of major effect QTLs between both types of populations, but new and additional QTLs were detected in the CSSL population. Although, three major effect QTLs for plant height were detected on chromosomes 1, 4, and 8 in the CSSL population, the latter two escaped detection in both RIL populations. Since this was observed for many traits, epistasis may play a major role for the phenotypic variation observed in weedy rice. High levels of shattering and seed dormancy in weedy rice might result from an accumulation of many small effect QTLs. Several CSSLs with desirable agronomic traits (e.g. longer panicles, longer grains, and higher seed weight) identified in this study could be useful for rice breeding. Since weedy rice is a reservoir of genes for many weedy and agronomic attributes, the CSSL library will serve as a valuable resource to discover latent genetic diversity for improving crop productivity and understanding the plant domestication process through cloning and characterization of the underlying genes.

## Introduction

Weedy rice (*Oryza sativa*), commonly known as red rice, is an annual weed of the same genus and species as cultivated rice, characterized by high genetic flexibility and phenotypic plasticity [1]. It is prevalent in most rice growing states in the southern United States, Europe, Central and South America where no wild or weedy relatives are present in natural habitat. The evolution of the weedy rice ecotypes prevalent in USA is still a mystery [2]. Weedy rice of the southern U.S. rice belt is genetically diverse and might be closely related to *O*. *sativa ssp*. *japonica*, *O*. *rufipogon*, and *O*. *nivara* [3]. Molecular marker studies indicated a possible origin of U.S. weedy rice from hybridization between Asian cultivated rice and the wild ancestor of rice, *Oryza rufipogon* [4, 5]. Although there have been attempts to analyze many traits contributing to its weedy behavior [6–10], weedy rice has yet to be explored in a systematic way to provide insights into its evolution. Weedy rices exhibit a high degree of seed dormancy and seed shattering, which are crucial for their survival and persistence in a rice field. The ability of weedy rice to easily hybridize with closely related cultivated rice facilitates synthesis of unique genetic materials to study the genetic mechanisms associated with weedy rice evolution and the domestication process.

Compared to the earlier years of continued success in rice breeding, improving and stabilizing rice productivity has been a recent challenge due to limited variability in available rice germplasm. The domestication process played a major role in reducing genetic variability by 50–60% in cultivated rice compared to wild rice [11]. Therefore, introgression of superior alleles from diverse sources—including weedy rice—will be required to widen the gene pool of cultivated rice to breed cultivars with improved yield, quality, and stress tolerance [12]. U.S. weedy rice, adapted to southern U.S. rice growing areas, is a great reservoir of a wide range of useful genes (e.g. rapid seedling growth, higher root mass and deeper root, seed and seedling resistance to pathogen attack) [13]. An organized effort is needed to discover such valuable genes to boost rice productivity.

Chromosome segment substitution lines (CSSL) are a series of near-isogenic lines (NILs) containing the fragments of the whole donor genome [14]. These are developed by repeated backcrossing, in conjunction with molecular markers, to define and track the introgressed chromosome fragments in each advancing backcross or selfed generation [15]. Such CSSLs not only detect QTLs that escape detection in commonly used mapping populations [16], but also allow delimitation of these QTLs to a smaller region on the genome using a series of overlapping CSSLs [17]. These libraries are important reagents for discovery and characterization of useful genes of wild germplasm [14].

There are several advantages that can accrue from this CSSL mapping population: (a) it can be used as a permanent mapping population for mapping traits with more precision and higher efficiency; (b) the introgression lines will allow determination of the effect of introgressed fragments in various genetic backgrounds and different environments, and distinguish between additive and dominance effects; (c) it will allow investigations of interactions between QTLs, which are difficult to evaluate in traditional mapping populations; (d) the introgression lines will be useful genetic materials for application of functional genomics tools to discover novel genes by focusing on differences in gene expression or protein expression to small chromosomal regions; and (e) the introgression lines can be used as varieties if any desirable trait can be precisely introgressed to the recipient cultivar. The CSSL libraries have been developed for several wild species of rice [18], and their utility has been demonstrated through successful mapping, cloning, and discovery of gene interactions for important agronomic traits [19, 20].

In this study, we report the development and characterization of a CSSL library of a U.S. weedy rice accession ‘PSRR-1’ with the genome-wide coverage in a U.S. rice cultivar ‘Bengal’ background. In addition, we mapped QTLs for several agronomic and domestication traits in two RIL populations involving the same weedy rice accession [21, 22] and compared the results with that from the CSSL population to determine the level of congruence between both populations. Since weedy rice harbors genes for both weedy and agronomically important traits, the utility of this CSSL library for QTL identification, fine mapping, map-based cloning, and gene interaction studies was discussed.

## Materials and Methods

### Plant materials and CSSL development

**Fig 1:**
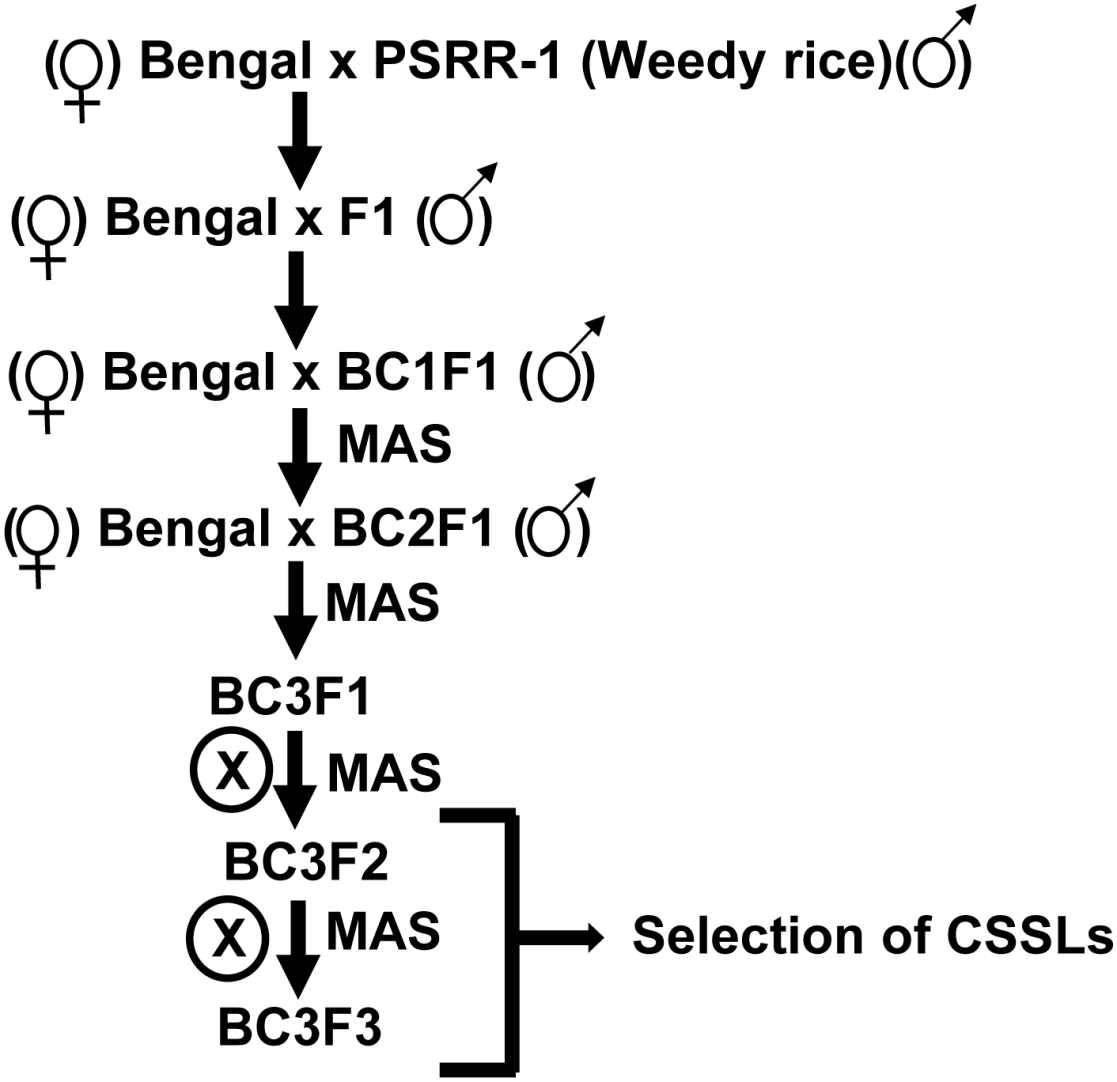
Breeding scheme for the development of the chromosome segment substitution lines (CSSLs). The CSSLs were developed from the weedy rice accession PSRR-1 in the rice cultivar ‘Bengal’ background. MAS: Marker assisted selection.

The plant materials for developing the CSSLs included the rice cultivar ‘Bengal’ and a weedy rice accession ‘PSRR-1’, which were used as recurrent parent (RP) and donor, respectively. Bengal is a high-yielding, medium grain rice cultivar released by the Rice Research Station of the Louisiana Agricultural Experiment Station, USA. PSRR-1 was purified by self-pollination and single plant selection for two generations from a mixture of weedy rice seeds collected from the South Farm, LSU Agricultural Center Rice Research Station located at Crowley, Louisiana. It possesses light green pubescent leaves, vigorous growth, long auricles and ligules, straw-hulled medium grains, and open panicles with few grains. PSRR-1 is extremely susceptible to shattering, and has a high intensity of both hull and pericarp dormancy compared with Bengal. Following the initial cross using Bengal as female, backcrossing to Bengal was performed three times, followed by two generations of selfing to develop a BC_3_F_3_ population for selection of homozygous CSSLs (Fig 1). Two RIL populations developed from the crosses, Bengal x PSRR-1 (BR-RIL) and Cypress x PSRR-1 (CR-RIL) [21, 22], were used for QTL mapping of various agronomic and domestication traits. The BR-RIL population consisted of 198 lines in the F_7:8_ generation, and the CR-RIL population had 174 lines in the F_8:9_ generation. Cypress is a high-yielding, long grain rice cultivar released by the Rice Research Station of the Louisiana Agricultural Experiment Station, USA.

### Molecular marker analysis

Fresh leaf tissues were collected from the 4-week old seedlings and DNA was isolated from each individual in each backcross generation and parents following the procedure of Tai and Tanksley (1990) [23]. One hundred sixty-one polymorphic simple sequence repeat (SSR) markers, uniformly distributed over the whole rice genome, were selected from the published linkage map with an average marker density of 6.6 cM [22] for tracking the donor weedy rice segments in each backcross generation. Primers were synthesized by Alpha DNA, Canada using the available primer information (http://www.gramene.org/microsat/RM_primers.html), and SSR analysis was carried out following Subudhi et al. (2012) [21]. The thermal profile was as follows: an initial denaturation step of 4 min at 94°C, followed by 40 cycles of 1 min denaturation at 94°C, 1 min annealing at 50°, 55°, or 60°C (depending on the individual SSR marker) and a 2 min extension at 72°C. After cycling, a final extension time of 10 min at 72°C was followed. The PCR products were separated on 4.5% superfine resolution agarose (Amresco) gels and visualized under UV light after ethidium bromide staining.

The linkage map developed in the same cross Bengal x PSRR-1 [21] was used as a reference to track the donor segments, as well as to determine the size of donor segments. In each backcross generation, the whole population was genotyped with selected SSR markers, and the marker profile of each line was recorded systematically. Specifically in each plant, the markers in the homozygous and heterozygous condition were listed. Only markers that were in the heterozygous condition in the previous generation were used for genotyping. Plants with a higher similarity with the recurrent parent were selected for the next round of backcrossing. This process was repeated in the BC_3_F_2_ and BC_3_F_3_ generation for selection of homozygous CSSLs. A core set of 74 CSSLs containing weedy rice segments was selected.

### Phenotypic evaluation

Both RIL populations along with their parents were grown at the Louisiana State University AgCenter Central Research Station in Baton Rouge, LA (30°20’51” N, 91°10’14” W) during spring 2009. Each line was sown in a single 2-meter row of 20 plants with row to row spacing of 20 cm. Standard cultural management procedures were followed throughout the season [24]. Five plants randomly sampled from each line were tagged, and measurements were taken on plant height (PH), panicle length (PL), flag leaf length (FLL), flag leaf width (FLW), grain length (GL), grain width (GW), and thousand grain weight (TGW).

The CSSL population, consisting of 74 lines in the BC_3_F_4_ generation, was grown at the Louisiana State University AgCenter Central Research Station in Baton Rouge, LA (30°20’51” N, 91°10’14” W) during spring 2011. The planting details for the CSSL population were the same as described above for the RIL populations. Observations were recorded on 10 random plants for the above seven agronomic traits, and five plants were randomly sampled for taking measurements on seed dormancy and seed shattering. Seed dormancy (SD) was evaluated in each CSSL and parents following the procedure of Subudhi et al. (2012) [21]. From each of the five sampled plants, three sets of 30 intact seeds with hulls were placed in petri plates lined with one layer of germination paper (Anchor Paper Co.) wetted with sterile distilled water and then placed in darkness at 30°C in an incubator. Observations were taken at 7 and 14 d after imbibition. Splitting of the hull by the emerging radicle was used as the criterion for visible germination. The number of seeds germinated was expressed as a percentage. Germination percent (GermARS) was arcsine transformed for data analysis. For seed shattering (SH), the breaking tensile strength (BTS), which is defined as the weight required to release a seed from the pedicel, was measured using a digital force gauge (Imada, Northbrook, IL) [22]. The digital force gauge was suspended from a stand with panicles attached to it. Individual seeds from the panicle were detached by holding the seed with a clip, and the peak measurement on grain removal was recorded. Ten readings of BTS values were recorded for each sampled plant.

### Data analysis

The length of substituted chromosome segments in CSSLs was estimated based on graphical genotypes [25]. If two adjacent markers showed the same allelic composition, it was assumed that the chromosome region was composed entirely of the marker genotype. If two consecutive markers showed different alleles along the chromosome of an individual, the interval was divided equally among both the markers. These estimates disregard the possibility of double recombinants within that interval. Marker orders were extrapolated from the linkage map developed from the RIL population of the cross Bengal x PSRR-1 [21]. The number and size of the chromosomal fragments based on total map length were estimated following Eshed et al. (1992) [26], and each of the backcross progeny was graphically characterized for the introgression of the weedy rice fragment(s). ‘CSSL finder’, which is a graphical genotyping program run in Microsoft Excel for managing introgression lines (http://mapdisto.free.fr/CSSLFinder/), was used for selection of a core set of CSSLs covering the weedy rice genome.

The presence of QTLs was inferred when there was a significant difference between the means of each CSSL and the recurrent parent using the Dunnett’s t-test at P<0.01. Standard analysis of variance and Dunnett’s test were conducted in SAS [27]. When QTLs were detected on overlapping chromosome segments in multiple CSSLs, their location was narrowed down to smaller regions using the principle of substitution mapping [17], in which meiotic recombinants were used to fine map the QTLs previously localized to chromosomal regions. The additive effect of each QTL was estimated as half the difference between trait mean of the CSSL and the trait mean of the recurrent parent [16].

Quantitative trait loci mapping in the RIL mapping populations was conducted using the composite interval mapping (CIM) procedure implemented in QTL Cartographer version 2.5 [28]. In the CIM procedure, a forward-backward regression procedure was followed with walk in speed of 1.0 cM. Threshold logarithm of the odds (LOD) values for CIM were calculated based on 1000 permutations (P < 0.01). In addition to the significant QTLs, the QTLs identified at LOD 2.5 were included as suggestive QTLs. The total phenotypic variation explained by all putative QTLs was estimated by fitting a model in the multiple interval mapping procedure of QTL Cartographer. The identified QTLs were compared with the results from single marker analysis. The QTLs were named in the same way as in our earlier studies [21, 22]. For example, *qSD1* ^*BR*^ and *qSD1* ^*CR*^ indicated seed dormancy QTL located on chromosome 1, but detected in the BR-RIL and CR-RIL, respectively.

## Results

### Development of a CSSL population with genome-wide coverage

Hybrids derived from the cross between Bengal and PSRR-1 were backcrossed consecutively for three generations with Bengal as the female parent to develop the CSSLs of the weedy rice accession PSRR-1. One hundred BC_1_F_1_ plants were genotyped with SSR markers, and 65 plants were selected for further backcrossing. In the BC_2_F_1_, 365 plants were genotyped, and 62 plants were selected for backcrossing to generate a BC_3_F_1_ population. Genotyping was done on 188 BC_3_F_1_ plants, and 128 plants were selected for selfing. From each of the selfed 128 BC_3_F_1_ plants, ten plants were grown. In total, 1280 BC_3_F_2_ plants were genotyped, and 112 plants were selected and selfed for the targeted donor segments covering the whole PSRR-1 genome. Preference was given to plants with homozygous weedy rice segments with a minimal number of heterozygous segments. The selfed progeny of those BC_3_F_2_ plants were grown and genotyped again for selecting the homozygous CSSLs. One hundred-sixty-one polymorphic SSR markers, which were previously placed on the RIL linkage map from the same cross [21], were used in marker assisted selection.

### Characterization of the CSSL population

**Fig 2:**
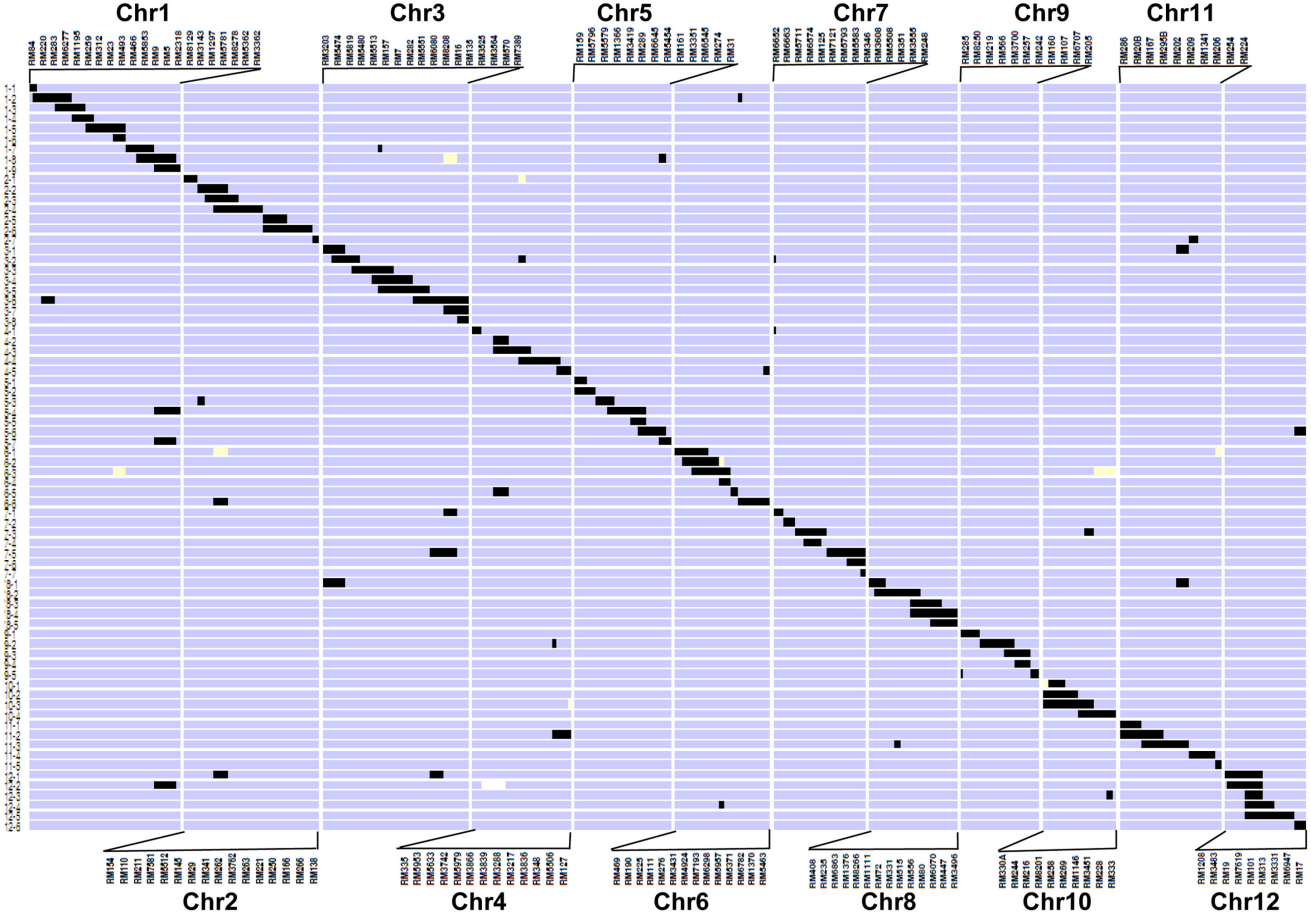
Graphical genotypes of the 74 chromosome segment substitution lines (CSSLs) of the donor U.S. weedy rice accession PSRR-1. The CSSLs are arranged vertically in order of their substituted chromosome segments. The regions in black represent homozygous regions for PSRR-1 alleles; the light blue regions indicate regions homozygous for recurrent parent Bengal alleles. The white regions are heterozygous segments.

Although there were 112 CSSLs, 74 CSSLs could provide coverage for the complete weedy rice genome (Fig 2). The graphical genotypes of this CSSL library revealed 99% coverage of the donor PSRR-1 genome, except for a small segment in the short arm of the chromosome 4 (defined by RM5633). Details of each CSSL with regards to number, as well as size of homozygous and heterozygous introgressed weedy rice segments, are given in S1 Table and S1 Fig The length of introgressed donor segments was estimated using the map positions of the molecular markers on the linkage map developed from the same cross Bengal x PSRR-1 [21].

**Fig 3:**
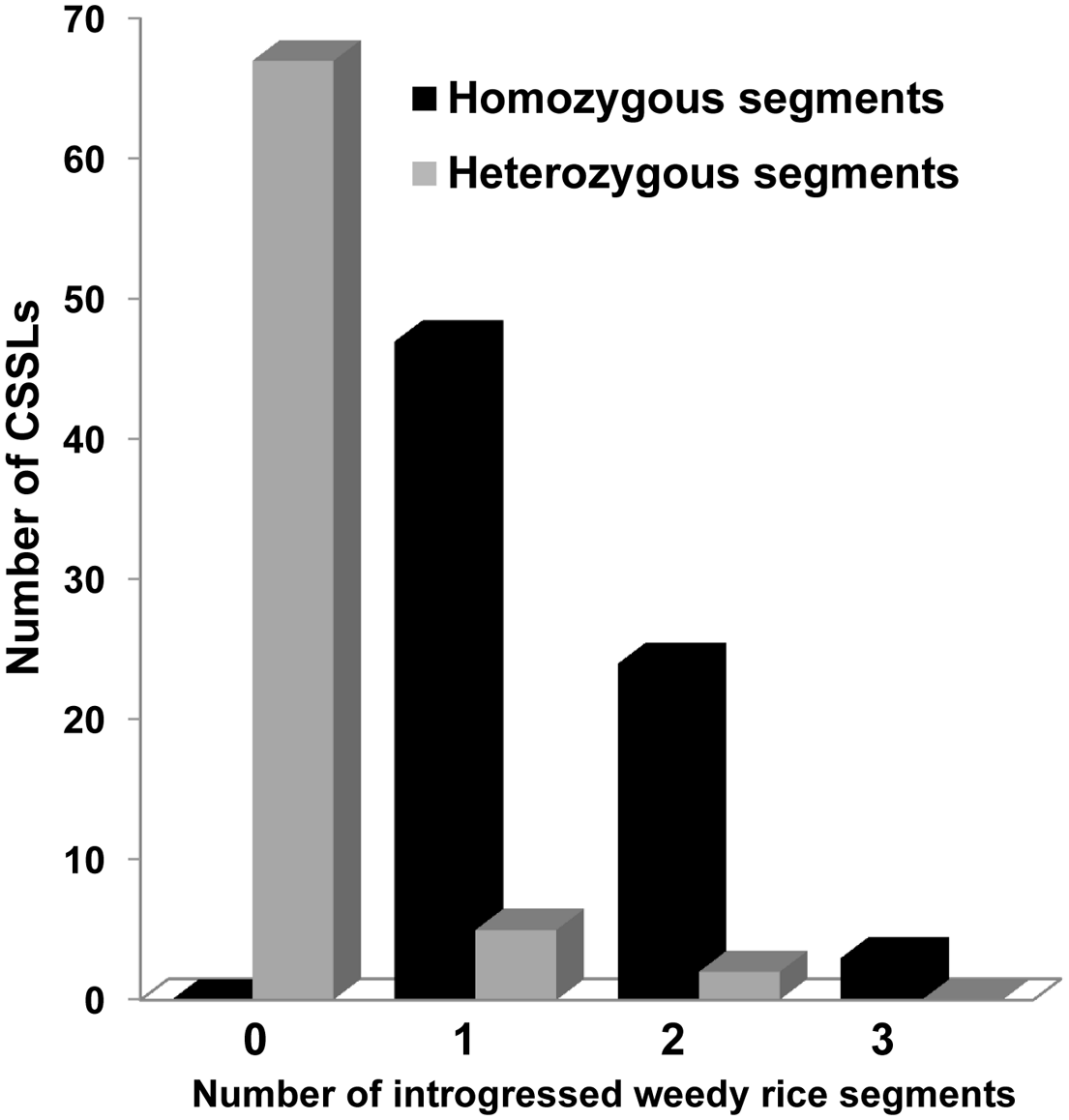
Frequency distribution of introgressed homozygous (black) and heterozygous segments (grey) in the weedy rice CSSL population.

**Fig 4:**
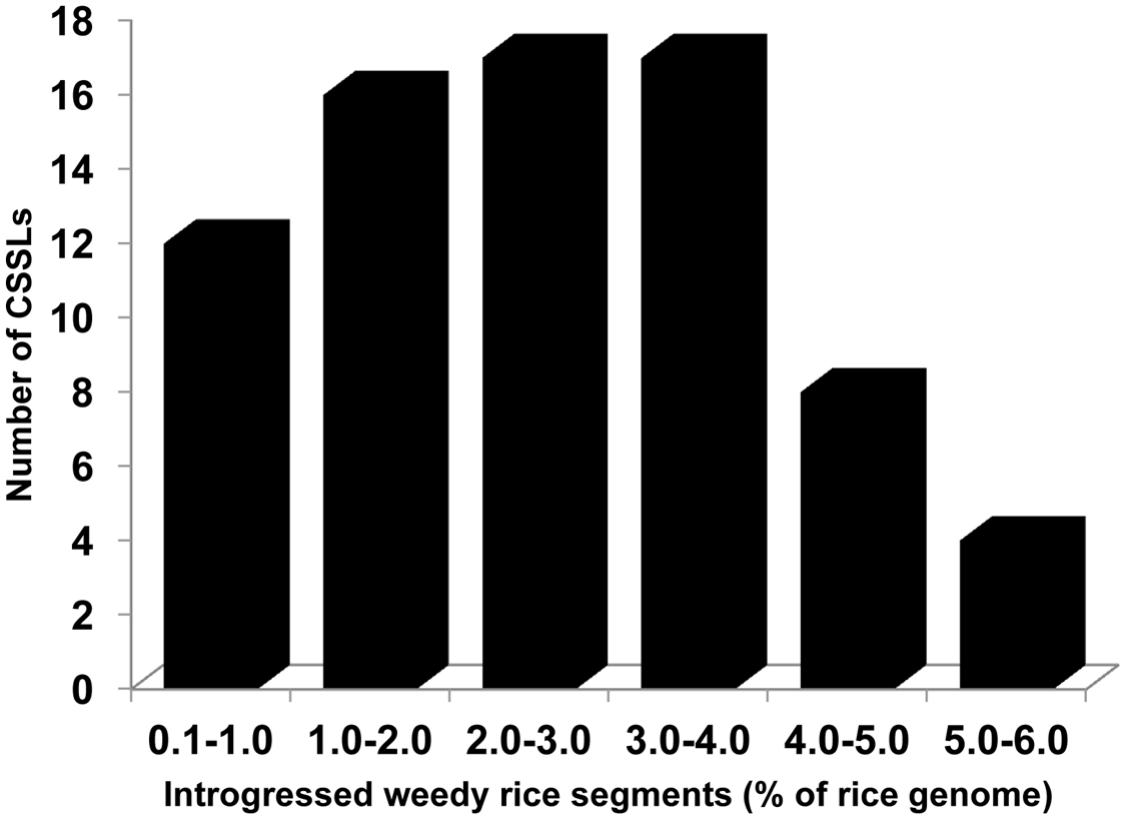
Frequency distribution of the donor segment length (% of the rice genome) in the weedy rice CSSL population.

There were 104 donor segments in total with an average of 1.4 introgressions per CSSL. The average size of an introgressed segment in each chromosome was 35.4 cM and 1.4 cM for homozygous and heterozygous segments of the donor genome, respectively. The quality of the CSSLs could be ascertained from the number and amount of introgressed donor segments (Figs 3 and 4). Only seven CSSLs harbored heterozygous segments. The number of donor segments ranged from 1–3 with 64% of CSSLs having single homozygous donor segments. The substituted weedy rice segments in each CSSL were 2.8% of the rice genome with a range of 0.4 to 5.6% and 62 CSSLs had 4% or less of the introgressed weedy rice genome.

### Phenotypic variation in parents and mapping populations

**Fig 5:**
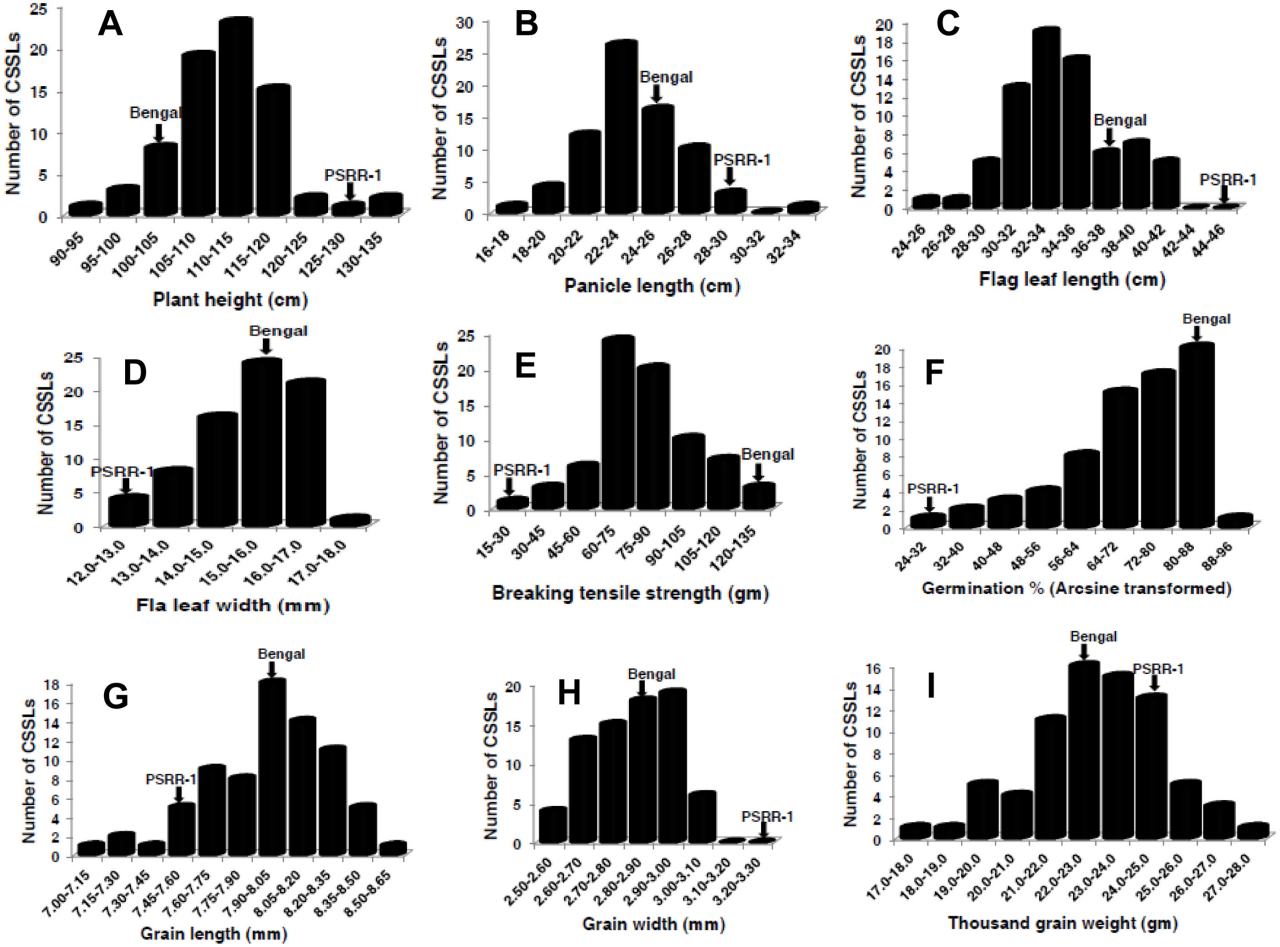
Frequency distribution for seven agronomic and two domestication traits in the weedy rice CSSL population. Mean phenotypic values of both parental lines are indicated by arrows. (A) Plant height; (B) panicle length; (C) flag leaf length; (D) flag leaf width; (E) breaking tensile strength; (F) germination% (arcsine transformed); (G) grain length; (H) grain width; (I) thousand grain width (I).

Data on seven agronomic traits (PH, PL, FLL, FLW, GL, GW, and TGW) and two domestication traits (SH and SD) for the parents and mapping populations (Table 1; Fig 5; S2 and S3 Figs) revealed a significantly reduced range of trait means for most of the traits in the CSSL population compared with the RIL populations. The mean trait values for the CSSL population were closer to those of the recurrent parent ‘Bengal’. Phenotypic variation revealed a normal distribution, but spread on both ends of the distribution beyond the parental values indicated transgressive variation for all seven agronomic traits in all populations. Weedy rice plants were taller with longer panicles, higher grain width, and higher thousand grain weight than both cultivars. Flag leaf length was longer in weedy rice but flag leaf width was more in Cypress. Cypress has longer grains compared to Bengal and weedy rice accession PSRR-1.

**Table 1:** Mean values for the agronomic and domestication traits in the parents, RILs, and CSSLs.

Trait	Bengal	PSRR-1	Cypress	BR-RILs^£^		CR-RILs^£^		BR-CSSLs^£^	
	Mean	Mean	Mean	Mean	Range	Mean	Range	Mean	Range
PH (cm)	105.1	128.1	75.2	99.9	50.2–166.0	91.8	47.1–167.6	110.2	91.2–131.1
PL (cm)	25.2	29.7	20.2	23.4	5.8–37.3	22.2	13.2–31.7	23.5	17.4–33.9
FLL (cm)	37.0	44.9	27.7	ND^¥^	ND	29.5	15.3–56.1	33.8	25.2–42.0
FLW (mm)	15.7	12.0	13.3	ND	ND	14.0	9.0–24.0	15.2	11.8–17.8
SH or BTS (gm) ^§^	5.0	9.0	7.0	7.0	3.0–9.0	6.5	2.3–9.0	83.2	29.3–222.9
GermARS	97.2	24.2	56.8	18.2	0.5–69.2	40.6	0.5–90.0	83.1	19.9–99.6
GL (mm)	7.9	7.5	8.3	8.0	6.6–9.3	8.3	6.2–9.9	7.9	7.2–8.6
GW (mm)	2.9	3.3	2.4	3.0	2.3–3.6	3.0	2.0–4.6	2.8	2.5–3.3
TGW (gm)	22.8	24.5	22.9	21.7	14.2–28.8	23.1	15.2–36.0	23.2	17.9–27.6

PH, plant height; PL, panicle length; FLL, flag leaf length; FLW, flag leaf width; SH, seed shattering; BTS, breaking tensile strength; GermARS, arcsine transformed value of germination%; GL, grain length; GW, grain width;

TGW, thousand grain width.

^¥^ND: Data not available

^§^ In both RIL populations and parents, SH was scored in a scale of 1–9 and BTS was measured in CSSLs.

^£^BR and CR correspond to Bengal x PSRR-1 and Cypress x PSRR-1 crosses, respectively.

Both parents differed significantly from PSRR-1 with respect to seed shattering and seed dormancy, and both RIL populations showed transgressive segregation for both traits [21, 22]. However, in the CSSL population, the distribution for BTS and SD was within the ranges of both parents (Fig 5).

### QTL mapping of agronomic traits in both RIL populations

The QTLs for SD and SH were reported earlier for both RIL populations [21, 22]. Here, we report the results for the agronomic traits that were subjected to QTL analysis using composite interval mapping. In both RIL populations, a wide range of variation was observed with respect to the magnitude of additive effects and percentages of the phenotypic variation explained by these QTLs; both weedy rice and cultivated rice alleles contributed to increased trait values. Five to nine QTLs were detected for the traits in BR-RIL population, whereas the number of QTLs varied from 1 to 7 in CR-RIL population (Tables 2 and 3; S4 Fig). The QTLs were spread over 9 chromosomes with the exception of chromosomes 9, 10, and 11.

**Table 2:** Quantitative trait loci for five agronomic traits detected in the RIL population developed from the cross Bengal x PSRR-1 using a composite interval mapping procedure.

Trait	QTL	Marker Interval	Position^a^	LOD Value	Additive effect^b^	PVE(%)^c^	DPE^d^
Plant height (PH)	*qPH1^BR^*	RM8278-RM8134	129.9	44.19	-20.410	50.7	R
	*qPH3-1^BR^*	RM81-RM5628	12.9	3.49	-4.727	2.8	R
	*qPH3-2^BR^*	RM3564-RM570	149.2	2.71	4.667	2.7	B
	*qPH4^BR^*	RM3217-RM3836	94.9	6.02	6.091	4.8	B
	*qPH5^BR^*	RM5311-RM6545	89.7	2.94	-4.316	2.3	R
	*qPH7^BR^*	RM214-RM5793	48.0	3.27	5.324	2.9	B
	*qPH12^BR^*	RM313-RM3331	63.6	2.63	-4.051	2.1	R
						74.5^e^	
Panicle length (PL)	*qPL1^BR^*	RM8278-RM8134	131.9	5.76	-1.141	9.9	R
	*qPL2^BR^*	RM29-RM341	55.3	4.49	-0.899	6.1	R
	*qPL3^BR^*	RM81 -RM5628	14.9	5.30	-1.002	7.3	R
	*qPL4^BR^*	RM3217-RM3836	91.9	2.81	0.710	4.0	B
	*qPL8^BR^*	RM515-RM556	63.8	4.89	-1.036	7.1	R
						32.1 ^e^	
Grain length (GL)	*qGL1^BR^*	RM5781-RM8278	121.9	5.07	0.013	6.0	B
	*qGL2^BR^*	RM1367-RM13910	104.0	3.09	0.009	3.7	B
	*qGL3^BR^*	RM3203-RM3372	2.0	6.93	-0.014	8.2	R
	*qGL4^BR^*	RM5506-RM127	114.6	5.39	0.013	6.9	B
	*qGL5^BR^*	RM3419-RM289	31.9	4.52	0.012	5.6	B
	*qGL6^BR^*	RM3431-RM4924	44.9	5.05	0.012	5.7	B
	*qGL7^BR^*	RM3608-RM5508	67.0	2.71	-0.009	3.3	R
	*qGL12-1^BR^*	RM1208-RM3483	0.0	2.74	-0.009	3.0	R
	*qGL12-2^BR^*	RM5479-RM28661	81.8	4.54	1.013	6.0	B
						46.3 ^e^	
Grain width (GW)	*qGW2^BR^*	RM145-RM29	51.4	7.58	0.0084	10.6	B
	*qGW4^BR^*	RM3288-RM3217	80.1	5.33	-0.007	7.6	R
	*qGW5-1^BR^*	RM5579-RM1366	10.7	3.81	-0.006	4.5	R
	*qGW5-2^BR^*	RM3419-RM289	32.9	13.73	-0.012	20.1	R
	*qGW5-3^BR^*	RM7568-RM161	66.3	3.09	-0.006	3.8	R
	*qGW5-4^BR^*	RM274-RM31	102.9	3.26	0.006	4.2	B
	*qGW7^BR^*	RM22134-RM248	103.4	4.11	0.006	4.8	B
	*qGW12^BR^*	RM313-RM3331	59.6	3.40	0.006	5.4	B
						63.2 ^e^	
Thousand grain weight (TGW)	*qTGW2^BR^*	RM145-RM29	46.4	3.23	0.638	4.7	B
	*qTGW3^BR^*	RM3203–RM3372	3.0	4.48	-0.771	6.6	R
	*qTGW6^BR^*	RM3431–RM4924	47.9	2.64	0.616	4.1	B
	*qTGW7^BR^*	RM6663-RM5752	6.6	3.93	0.701	5.5	B
	*qTGW12^BR^*	RM5479-RM28661	82.8	7.96	0.996	11.7	B
						28.4 ^e^	

^*a*^ QTL peak position on the linkage map.

^*b*^ Additive effects of Bengal allele.

^*c*^ Phenotypic variation (%) explained by each QTL.

^*d*^ DPE, direction of phenotypic effect. B and R denote Bengal and PSRR-1 alleles increasing the phenotypic values, respectively.

^*e*^ Estimate of the total phenotypic variation explained by the QTLs from a multiple QTL model fit in QTL Cartographer [28].

**Table 3:** Quantitative trait loci for seven agronomic traits detected in the RIL population developed from the cross Cypress x PSRR-1 using a composite interval mapping procedure.

Trait	QTL	Marker Interval	Position^a^	LOD Value	Additive effect^b^	PVE(%)^c^	DPE^d^
Plant height (PH)	*qPH1-1^CR^*	RM84-RM283	5.0	3.21	5.519	3.2	C
	*qPH1-2^CR^*	RM7250-RM5362	144.0	29.98	-21.038	48.9	R
	*qPH4^CR^*	RM5503-RM348	83.7	5.37	8.201	5.5	C
	*qPH7^CR^*	RM3555-RM172	114.5	2.72	6.091	3.6	C
	*qPH10^CR^*	RM3311-RM8201	19.7	4.76	-7.808	5.9	R
	*qPH12-1^CR^*	RM7315-RM6296	15.2	2.74	5.653	3.2	C
	*qPH12-2^CR^*	RM1337-RM28424	69.2	2.74	-4.962	2.7	R
						72.8^e^	
Panicle length (PL)	*qPL1^CR^*	RM7250-RM5362	141.0	5.64	-1.737	10.6	R
	*qPL4^CR^*	RM348-RM5506	94.0	2.70	1.130	4.3	C
						14.3	
Grain length(GL)	*qGL5^CR^*	RM164-RM4674	73.0	3.22	-0.478	4.9	R
Grain width(GW)	*qGW3^CR^*	RM570-RM7389	173.9	3.98	-0.173	6.1	R
	*qGW7-1^CR^*	Rc-RM214	34.0	4.64	0.200	6.5	C
	*qGW7-2^CR^*	RM351-RM6810	79.2	4.46	-0.168	6.4	R
						10.6 ^e^	
Thousand grain weight (TGW)	*qTGW5^CR^*	RM164-RM4674	76.1	4.90	-2.065	12.9	R
Flag leaf length (FLL)	*qFLL1^CR^*	RM7250-RM5362	144.0	13.22	-4.309	22.8	R
	*qFLL6^CR^*	RM276-RM8225	56.3	3.06	-1.968	3.9	R
	*qFLL7^CR^*	RM6810-RM3555	104.1	7.91	3.627	16.1	C
	*qFLL11^CR^*	RM3428-RM229	65.2	3.67	1.953	4.3	C
	*qFLL12^CR^*	RM7315-RM6296	14.2	3.74	2.241	5.4	C
						49.4 ^e^	
Flag leaf width (FLW)	*qFLW3-1^CR^*	RM3203-RM3372	0.0	5.01	-0.068	6.9	R
	*qFLW3-2^CR^*	RM3513-RM3525	105.6	4.11	-0.069	7.1	R
	*qFLW4^CR^*	RM5503-RM348	84.7	7.33	0.106	12.1	C
	*qFLW12-1^CR^*	RM7315-RM6296	30.2	7.86	0.112	12.4	C
	*qFLW12-2^CR^*	RM6973-RM1337	50.8	3.45	-0.068	4.5	R
						31.6 ^e^	

^*a*^ QTL peak position on the linkage map.

^*b*^ Additive effects of Cypress allele.

^*c*^ Phenotypic variation (%) explained by each QTL.

^*d*^ DPE, direction of phenotypic effect. C and R denote Cypress and PSRR-1 alleles increasing the phenotypic values, respectively.

^*e*^ Estimate of the total phenotypic variation explained by the QTLs from a multiple QTL model fit in QTL Cartographer [28].

There were seven QTLs for plant height in each RIL population. One major QTL was identified on chromosome 1 with LOD scores of 44 and 30, explaining phenotypic variation of 51% and 49% in BR and CR populations, respectively. The weedy rice allele of this major QTL was responsible for increasing plant height in both populations. The remaining QTLs were minor effect QTLs with phenotypic variation ranging between 2% and 6%. The total variance explained by all QTLs in each population was around 75%.

For panicle length, five QTLs were identified on chromosomes 1, 2, 3, 4, and 8 in the BR population with a total phenotypic variance of 32%. The phenotypic variance explained by these QTLs varied from 4 to10%. Weedy rice alleles in four QTLs increased panicle length. In the CR population, only two QTLs, with contributions of 4% and 11% towards phenotypic variation, were identified for this trait. The weedy rice allele increased panicle length for the QTL with a large effect (*qPL1* ^*CR*^).

For grain length, grain width, and thousand grain weight, five to nine QTLs were mapped in the BR population compared to 1–3 QTLs in the CR population. In both populations, the phenotypic variance explained by a majority of QTLs was less than 10%, with exception for three major QTLs (*qGW2* ^*BR*^, *qGW5-2* ^*BR*^, and *qTGW12* ^*BR*^ *)* and one major QTL *(qTGW5* ^*CR*^) in BR and CR populations, respectively. In case of *qGW5-2* ^*BR*^, and *qTGW5* ^*CR*^, the weedy allele increased the trait values, but the cultivated rice alleles were favorable for *qGW2* ^*BR*^, and *qTGW12* ^*BR*^.

There were five QTLs for flag leaf length and flag leaf width in the CR population, with a range of phenotypic variance from 4–23% and LOD values of 3.0–13.0. Total phenotypic variances explained by all QTLs were 49% and 32% for the flag leaf length and flag leaf width, respectively. The two major QTLs for flag leaf length (*qFLL1* ^*CR*^ and *qFLL7* ^*CR*^) were localized on chromosomes 1 and 7, with the weedy allele responsible for increased leaf length in the former, while the Cypress allele accounted for the later. However, Cypress derived alleles were responsible for increasing flag leaf width in case of both major QTLs (*qFLW4* ^*CR*^ and *qFLW12-1* ^*CR*^).

### QTL mapping using the CSSL population

**Fig 6:**
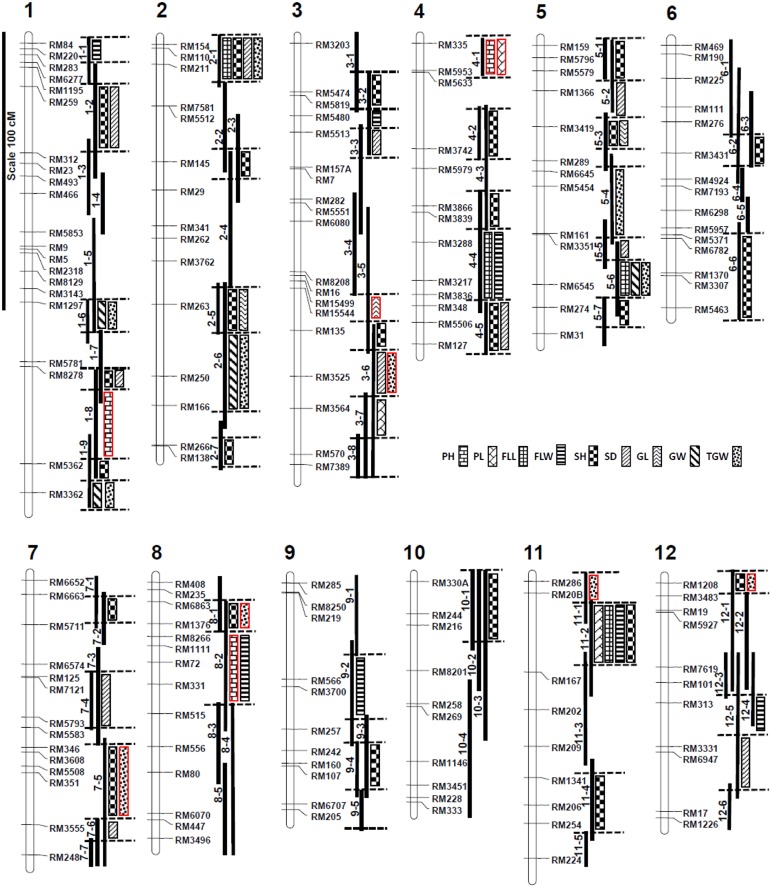
Chromosomal location of introgressed weedy rice segments and quantitative trait loci for seven agronomic and two domestication traits in weedy rice CSSLs. The linkage map developed in a recombinant inbred line (RIL) population from the cross between Bengal and PSRR-1 [21] was used to determine the substituted segments and coverage of rice genome in each CSSL. The bars to the right side of chromosomes indicate the substituted weedy rice chromosome segments in the CSSLs. The presence of a QTL is inferred when there is a significant difference between the means of each CSSL and the recurrent parent at p<0.01 using the Dunnett’s test. When multiple CSSLs with overlapping chromosome segments are significantly different from the recurrent parent for the trait values, the QTL location was narrowed down to smaller region using substitution mapping [17]. Bars with a red border indicate the PSRR-1 alleles responsible for increased trait values.

The mean values for each trait of the individual CSSL were compared with the recurrent parent, and QTLs were assigned to the introgressed region if the differences were significant at p <0.01 (Fig 6; S2 Table). If CSSLs with overlapping introgressions were significantly different from the recurrent parent, the QTL location was determined using substitution mapping [17].

**Fig 7:**
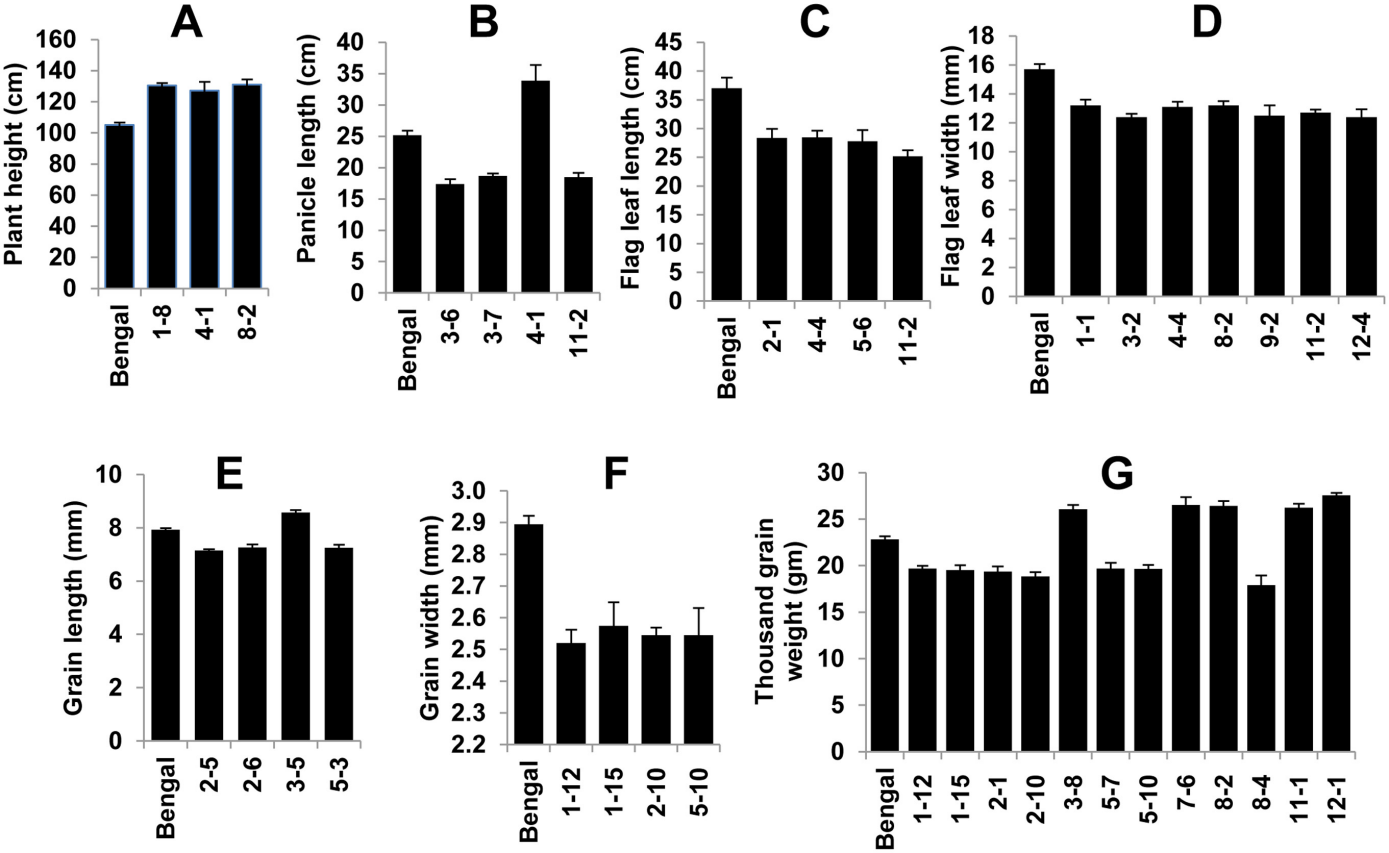
Chromosome segment substitution lines (CSSLs) that are significantly different for various agronomic traits compared to the recurrent parent Bengal’ at p < 0.01. (A) plant height; (B) plant length; (C) flag leaf length; (D) flag leaf width; (E) grain length; (F) grain width; (G) thousand grain weight. Bars indicate the mean values of traits±standard error. Data from the CSSLs with overlapping chromosome segments were used to narrow down the QTL regions for traits using substitution mapping [17].

Three major PH QTLs with similar levels of additive effects were identified on chromosomes 1, 4, and 8. Weedy rice alleles increased plant height to 127–131 cm (Fig 7). For panicle length, three QTLs with large effects were on chromosomes 3, 4, and 11. The weedy rice allele for the QTL on chromosome 4 increased panicle length but reduced it in QTLs on chromosomes 3 and 11 compared to Bengal (Fig 7). Four CSSLs had significantly smaller FLL compared with Bengal. Four QTLs for flag leaf length with similar levels of additive effects were localized on chromosomes 2, 4, 5, and 11. The weedy rice accession PSRR-1 had longer FLL compared with Bengal. But none of the CSSL had increased FLL. It was in fact decreased significantly in case of 4 CSSLs, and distribution of FLW was a little skewed toward wider leaves in the CSSL population (Fig 6). Flag leaf width was narrower in PSRR-1 than Bengal. But as expected, weedy rice introgression reduced FLW in seven CSSLs (Fig 7), and therefore 7 corresponding QTLs were localized on chromosomes 1, 3, 4, 8, 9, 11, and 12.

Although both Bengal and PSRR-1 were medium grained, Bengal grains were a little longer than PSRR-1. Three CSSLs with weedy rice introgressions from chromosomes 2 and 5 reduced grain length, and only one CSSL (3–5) had increased grain length (Fig 7). Weedy rice alleles increased grain length for the QTL on chromosome 3 and reduced grain length in case of QTLs on chromosomes 2 and 5. Grains were wider in PSRR-1 than Bengal. Four QTLs with a similar level of additive effects were identified for GW, and all four significant CSSLs had reduced grain width (Fig 7, S2 Table). Grain weight in weedy rice was slightly more than Bengal. Twelve CSSLs with introgressed segments from eight chromosomes were significantly different from Bengal with respect to TGW (S2 Table). The fact that seven CSSLs had lower TGW and 5 CSSLs had higher TGW suggested a contribution of both parental alleles towards increased TGW.

**Fig 8:**
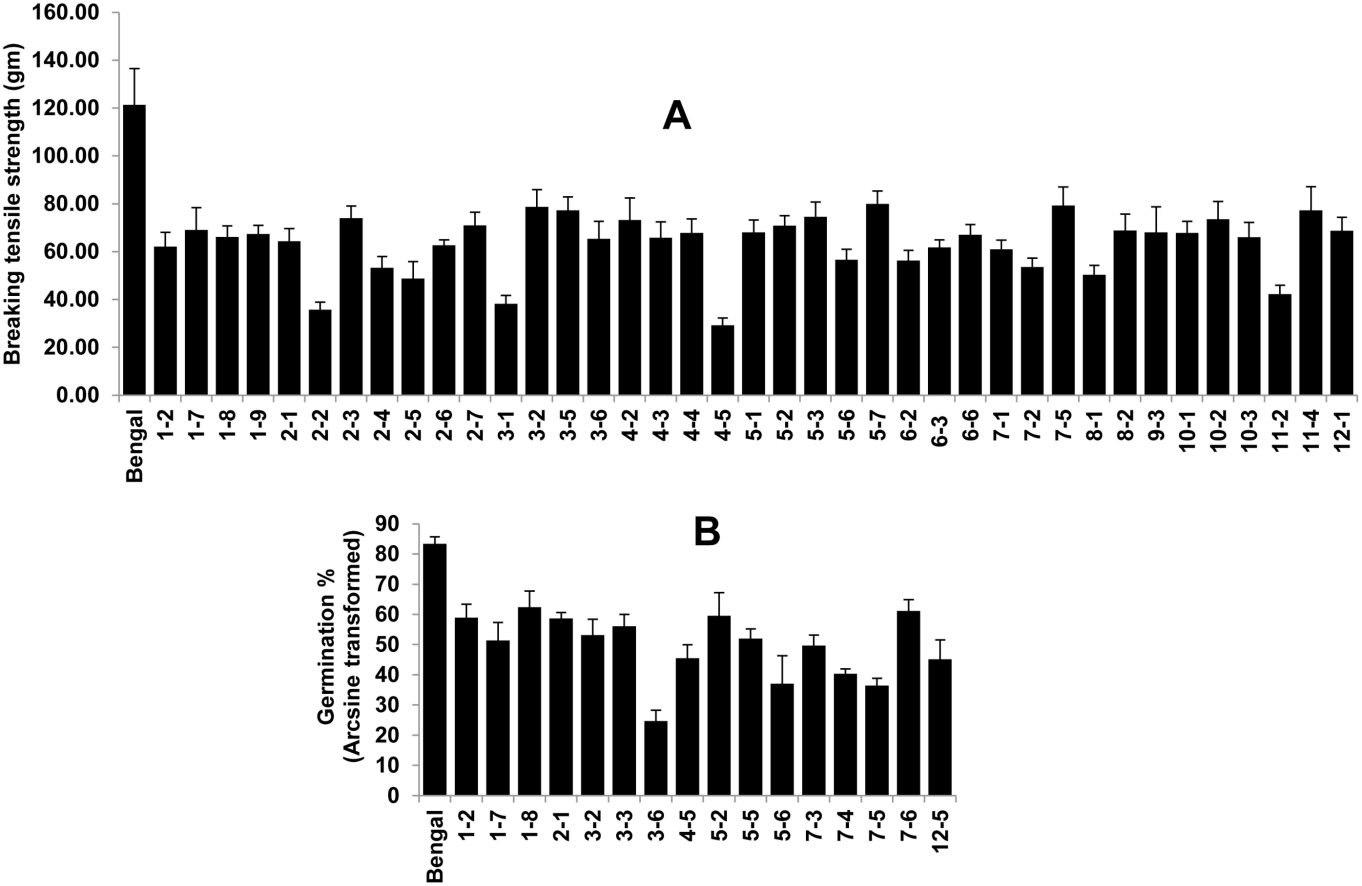
Chromosome segment substitution lines (CSSLs) that are significantly different for breaking tensile strength (A) and germination % (Arcsine transformed) (B) compared to the recurrent parent Bengal’ at p<0.01. Bars indicate the mean values of traits ± standard error. Data from the CSSLs with overlapping chromosome segments were used to narrow down the QTL regions for traits using substitution mapping [17].

The breaking tensile strength (BTS) value for PSRR-1 was negligible, i.e. closer to 0, whereas the BTS was around 120 gm for Bengal. Thirty nine CSSLs with weedy rice introgressions from all 12 chromosomes had reduced BTS (Fig 8), which suggested the involvement of multiple loci for seed shattering with both large and small effects. Among the CSSLs, CSSL4-5 has the lowest BTS followed by CSSL2-2, CSSL3-1, and CSSL11-2. Using these phenotypic data (S2 Table), 25 QTLs were mapped.

Sixteen CSSLs had significantly lower germination percentages than Bengal, and the PSRR-1 allele contributed toward seed dormancy in all these cases. CSSL3-6 has the lowest germination %. Eleven QTLs were mapped based on the germination data.

Strong positive correlations were observed between PH and PL, GL and TGW, and GW and TGW in all three populations (S3 Table). The FLL was significantly correlated to PL and GL in the CR-RIL and CSSL populations. The QTL locations of these correlated traits in both RIL populations overlapped in a majority of cases indicating the pleiotropy or linkage. When QTL locations for those correlated traits in the CSSL population were analyzed, the degree of concordance was reduced compared with the RIL population, but there was still considerable overlapping of QTLs of highly correlated traits, such as TGW and GW, FLL and FLW, PH and PL, and PH and FLL.

### Congruency of QTLs between RIL and CSSL population

The location and magnitude of variation explained by the QTLs, and the direction of the phenotypic effects, were examined for agronomic traits to determine the congruency in both RIL populations. Common SSR markers placed on both linkage maps allowed such comparisons (Tables 2 and 3; S4 Fig). There were 3 and 2 congruent QTLs for PH and PL, respectively. The QTL with a major effect, *qPH1* ^*BR*^, corresponded to *qPH1-2* ^*CR*^. The congruent minor effect QTLs for PH localized on chromosomes 4 and 12, were similar with respect to the direction of the phenotypic effect and the phenotypic variation explained by these QTLs. When compared with the QTLs detected in the CSSL population, only the major QTL on chromosome 1 could be detected. On the contrary, two major effect plant height QTLs detected on chromosomes 4 and 8 escaped detection in either RIL population. Both CSSL4-1 and CSSL8-2 showed similar plant height as CSSL1-8, which most likely harbored the green revolution gene *sd1* [29, 30].

In the case of PL, congruent QTLs on chromosomes 1 and 4 in both RIL populations were quite similar in magnitude. The weedy rice allele was responsible for increasing the PL in case of the former, whereas it was cultivated rice allele for the QTL on chromosome 4. None of the QTLs overlapped with the QTLs for PL in the CSSL population. For FLL, none of the QTLs could be validated in the CSSL population, but two QTLs for FLW, *qFLW4* ^*CR*^ and *qFLW12-2* ^*CR*^, were localized on the same chromosomal regions.

The *qGL2* ^*BR*^ and *qGL5* ^*BR*^ were in the same position as the QTLs in the CSSL population, and in both cases, the weedy rice allele decreased grain length. The QTL allele that increased grain length in CSSL3-5 could be useful for rice breeding. There was no correspondence between QTLs identified in the RIL and CSSL populations for grain width and thousand grain weight.

The major QTL for SH, corresponding to the *sh4* locus on chromosome 4, was in the identical position in both RIL populations [22] and the CSSL population. Three minor QTLs on chromosomes 2, 3, and 7 in the BR-RIL population and one minor QTL on the chromosome 6 in the CR-RIL population were also congruent with the QTLs for SH in the CSSL population. There were 11 QTLs for seed dormancy identified in the CSSL population; however, we could validate only the major QTL near the red pericarp locus on chromosome 7 and one minor QTL on chromosome 3, which were consistently identified in both RIL populations [21].

## Discussion

Realizing the importance of wild and weedy species diversity for crop improvement, different types of mapping populations have been generated for locating genes underlying complex traits of economic importance [12, 31]. During the past decade, the power and usefulness of CSSL populations in mapping, map-based cloning, and gene interaction studies have been demonstrated in numerous studies [18, 20, 32–35]. *Oryza rufipogon*, considered as the progenitor of cultivated rice, has been widely used in search for valuable alleles for genetic improvement and CSSLs have been generated and yield improving alleles have been identified [36–38]. Weedy rice is unique in many respects and is not considered a wild species despite its many conspicuous wild species characteristics, such as higher seed dormancy, seed shattering, and red pericarp. We report the development of a CSSL library of a straw-hulled U.S. weedy rice ecotype consisting of 74 lines in a cultivated rice background for genetic dissection of complex agronomic and domestication traits.

Both phenotypic variation and coverage of the donor genome in the CSSL population are critical to realize its potential in the context of both genetic dissection and usefulness for crop improvement [32]. Incomplete coverage of a donor genome in the CSSL population might miss some QTLs responsible for useful traits. The genome coverage in CSSLs of *Oryza rufipogon* reported earlier ranged from 68% to 99% [32, 36, 39, 40]. In the study of Tian et al. 2007 [36], although the donor genome coverage was low, the CSSLs had only 2% of the donor genome per line. Ten percent of the donor genome was missing in the CSSLs developed by Furuta et al. (2014) [39], whereas it was only 1% in the CSSLs of Tan et al. 2007 [32]. Recently, *O*. *rufipogon* and *O*. *meridionalis* were used for construction of two CSSL libraries in a cultivated rice background [40], which covered 77% and 98% of the donor genome, respectively. Low coverage of the donor genome [36, 40, 41] could be due to several factors such as genetic divergence, photosensitivity, sterility, gametophytic genes, or lack of marker-based selection for the donor genome. Although we observed considerable sterility and photosensitivity in this population, marker assisted selection using polymorphic SSR markers covering the whole rice genome in every backcross generation and growing of these backcross populations in a short day environment was helpful for successful development of this CSSL population with coverage of 99% of the donor genome.

When we compared the phenotypic variation between RIL and CSSL populations (Fig 6; S2 and S3 Figs), we found substantial narrowing of the phenotypic range for most traits in the later, which could be due to segregation of one or a few QTL regions compared with the F_2_ or RIL populations. The phenotypic variation resulting from epistatic interactions is hardly targeted for crop improvement by plant breeders due to the difficulty in manipulation. Therefore, the desirable variability due to additive genetic effects in a CSSL population could be immediately and easily usable in breeding programs.

Evaluation of CSSLs gives an accurate estimate of the gene effect due to the near-isogenic background. It eliminates the interaction of non-linked QTLs in determining the QTL locations. Development of both RIL and CSSL populations from the same cross could be helpful for genetic investigations [42]. Due to clear differences in the genetic profile of individual CSSLs, correlating a particular chromosomal region to phenotypic variation is much simplified in a CSSL population. For example, any significant difference in trait values between a CSSL and the recurrent parent could be attributed to the introgressed donor segment in that particular CSSL. Similarly, narrowing of the target chromosomal region for a particular trait could be possible by comparison of the phenotypic values of overlapping introgression lines [17]. The U.S. weedy rice accession PSRR-1 has been used earlier in two RIL populations for mapping QTLs for seed dormancy [21] and seed shattering [22], and QTLs for number of agronomic traits were mapped in this study. Therefore, a CSSL population coupled with both RIL populations will be a valuable permanent resource for genetic studies.

Differences in the mapping power of both types of populations became apparent upon comparison of results between the CSSL and RIL populations involving the same weedy rice accession PSRR-1 (Tables 2 and 3; S2 Table). Increased phenotypic variance observed in the RIL population compared with the CSSL population could be attributed to the increased level of transgression and epistatic interactions. Overall, more and different QTL regions were detected in the CSSL population compared with the RIL populations. This observation demonstrated its complementary nature in detection of QTLs with increased efficiency [42]. Variability in detected QTLs in terms of the position and effect in both populations was dependent on the trait. In case of plant height, seed shattering, and seed dormancy, the major QTLs identified in RIL populations on chromosomes 1, 4, and 7 (respectively) were validated in the CSSL population. Few new QTLs with large effects were detected for PH, PL, and TGW. The weedy rice has longer FLL and greater GW compared to Bengal, but none of the CSSLs had increased FLL or GW. In fact, some CSSLs had reduced FLL and GW (Fig 7). For seed shattering, the large number of QTLs with significant effects that were identified in CSSL population could be due to more precise determination of seed shattering by measuring the BTS in lieu of scoring on a scale of 0–9. A higher degree of SH observed on CSSL2-2 was due to the additional major SH locus-containing chromosome 4, harboring the *sh4* locus. The high shattering in weedy rice might result from accumulation of many small effect QTLs, which is in agreement with the conclusion of Subudhi et al. (2014) [22]. Similar logic can be applied to seed dormancy, where several CSSLs with significantly higher seed dormancy, compared with the recurrent parent, were observed. Since the CSSL3-6 showed high seed dormancy compared to the CSSL7-5 carrying the major QTL [21], it could be due to a major QTL not yet identified. Although the power of resolution in the RIL population is higher compared to the CSSL population, the reverse is the case with respect to detection of small effect QTLs [40]. The minor QTLs identified using traditional mapping populations are of little practical utility for crop improvement. On the contrary, CSSLs with small effect QTLs introgressed from wild or weedy donors in an adapted elite cultivar background could be useful for improving target traits through pyramiding of these QTLs in breeding programs.

The usefulness of the CSSL population lies in providing starting materials to initiate fine mapping and Mendelization of both large and small effect QTLs, which ultimately paves the path for map-based cloning of QTLs. Although CSSL libraries have been developed for a few wild rice species [32–33, 35, 36, 43, 44], no CSSL population has been developed for any weedy rice accession. Therefore, the CSSL population with genome-wide coverage reported in this study will be a valuable tool to elucidate the molecular basis of both weedy and agronomic traits.

CSSLs can be useful to study gene interactions. In this study, the major plant height QTL on chromosome 1 was detected in both RIL and CSSL populations. But two major QTLs on chromosome 4 and 8 identified in CSSL population escaped detection in RIL populations, suggesting epistatic interactions. Since this was observed for other traits (e.g. FLL and GW), epistasis may be playing a major role in trait evolution in weedy rice [45]. The CSSLs can also be used to infer QTL x Environment interactions [46] or impact of QTLs in different genetic backgrounds [47].

Wild and weedy rice can serve as a source for many agronomically important genes, which are not easy to discover by phenotypic assessment [48]. The CSSLs have been successfully utilized to discover new alleles for heading date [49], awns [35], blast resistance [50], green leafhopper resistance [51], and brown plant hopper resistance [37]. The CSSLs with desirable agronomic traits such as longer panicles, longer grains, and higher TGW reported here can be used as prebreeding materials to improve these traits through marker-assisted selection [52].

Despite the congruence of the SH and SD QTLs with the cloned genes *sh4* and *Sdr4* in both RIL populations [21, 22], the molecular basis of these traits [53, 54] is still elusive in weedy rice. Thus, the CSSLs with high seed shattering (4–5) and seed dormancy (7–5) would be appropriate to revisit these issues. Taller plant stature in the weedy rice accession PSRR-1 compared to the cultivars, Bengal and Cypress, is most likely due to a functional GA20-oxidase gene required for gibberellin biosynthesis (Os01g66100) [29, 30]. Identification of two CSSLs (4–1 and 8–2) carrying major effect QTLs would be useful to further investigate the genetic architecture of plant height.

## Conclusions

This successfully generated CSSL population with genome wide coverage will be a powerful tool for broadening the genetic pool of breeding materials to improve rice productivity. Additionally, it will facilitate large scale gene discovery, gene interaction, and functional genomics studies. It provides an alternative platform to map and validate the known genes and QTLs for understanding the genetic complexity of both agronomic and domestication traits with special emphasis on small effect QTLs. Involvement of multiple genes and epistasis was inferred for the traits evaluated in this study. The CSSLs harboring both minor and major QTLs provide ready-made resources for fine mapping and map-based cloning projects [55]. We demonstrated that useful QTLs escape detection in RIL mapping populations. Therefore, simultaneous analysis of both mapping populations would be helpful to capture maximum variability for genetic improvement.

## Supporting Information

S1 Fig Length of introgressed segments (homozygous and heterozygous) in each CSSL for all 12 chromosomes. (TIF)

S2 Fig Frequency distribution of the RILs for five agronomic traits (plant height, panicle length, grain length, grain width, and thousand grain weight) in BR-RIL population. (TIF)

S3 Fig Frequency distribution of the RILs for seven agronomic traits (plant height, panicle length, flag leaf length, flag leaf width, grain length, grain width, and thousand grain weight) in CR-RIL population. (TIF)

S4 Fig Map location of QTLs for seven agronomic and two domestication traits in the CSSL population and recombinant inbred line populations. The RIL populations were developed from the cross Bengal × PSRR-1 (BR-RIL) and the cross Cypress × PSRR-1 (CR-RIL). The CSSL population was developed using the weedy rice accession PSRR-1 as a donor in a rice cultivar ‘Bengal’ background.
(TIF)

S1 Table Summary of the introgressed weedy rice PSRR-1 segments and genome coverage in individual chromosome segment substitution lines developed in Bengal background. (PDF)

S2 Table Quantitative trait loci, additive effects, and direction of phenotypic effects for plant height, panicle length, flag leaf length, flag leaf width, breaking tensile strength, germination % (arcsine transformed), grain length, grain width, and thousand grain weight derived from the evaluation of the CSSLs and the recurrent parent Bengal. (PDF)

S3 Table Phenotypic correlation (Pearson correlation coefficient) among the traits in weedy rice CSSL population (n = 74) and RIL populations. (PDF)
