# Supplementary figures and images for: A Chromosome Segment Substitution Library of Weedy Rice for Genetic Dissection of Complex Agronomic and Domestication Traits
Source: PLoS One. 2015 Jun 18;10(6):e0130650. doi: 10.1371/journal.pone.0130650 (PMC4472838; doi:10.1371/journal.pone.0130650)

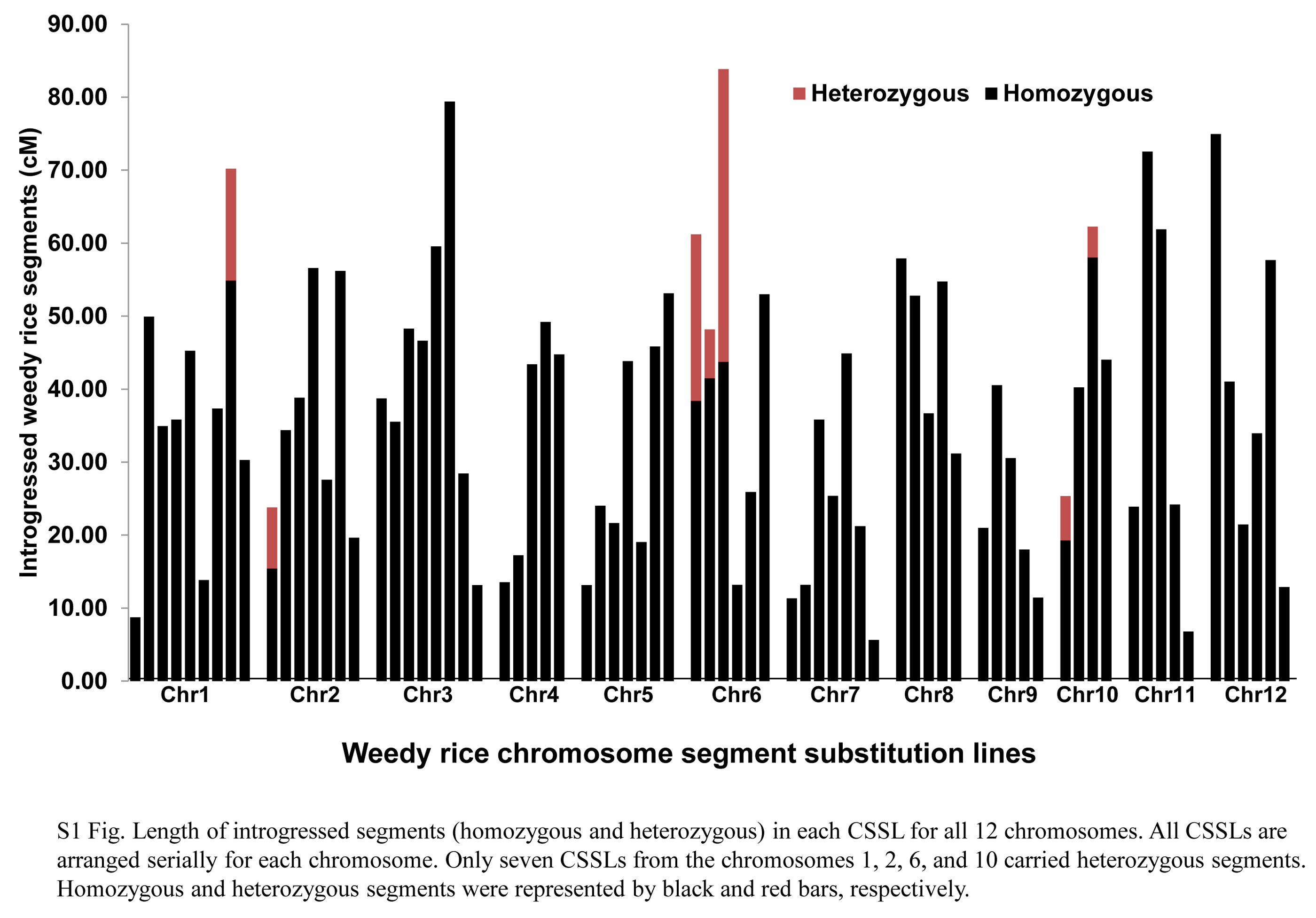

Supplement: S1 Fig — (TIF) [file pone.0130650.s001.tif]

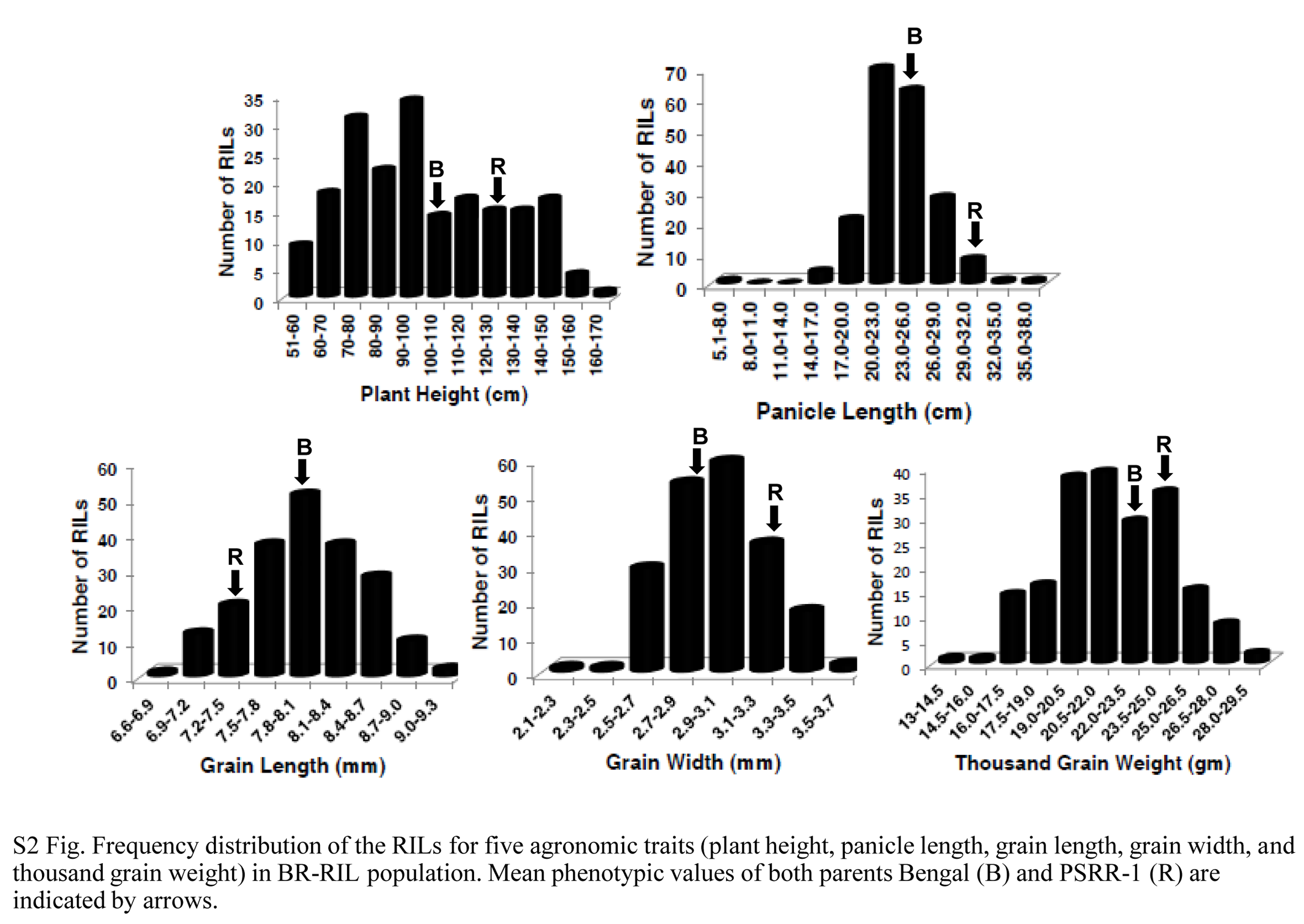

Supplement: S2 Fig — (TIF) [file pone.0130650.s002.tif]

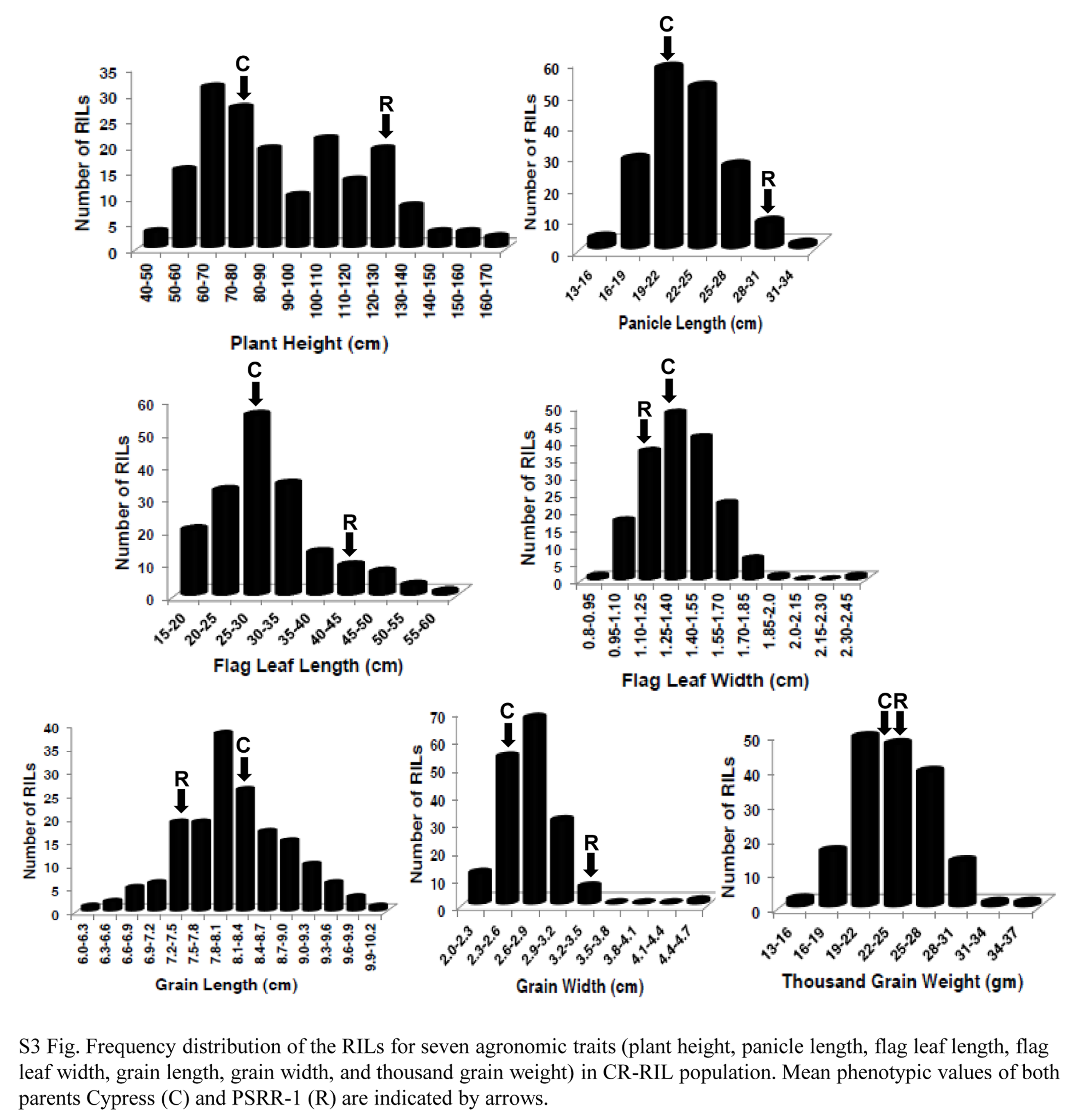

Supplement: S3 Fig — (TIF) [file pone.0130650.s003.tif]

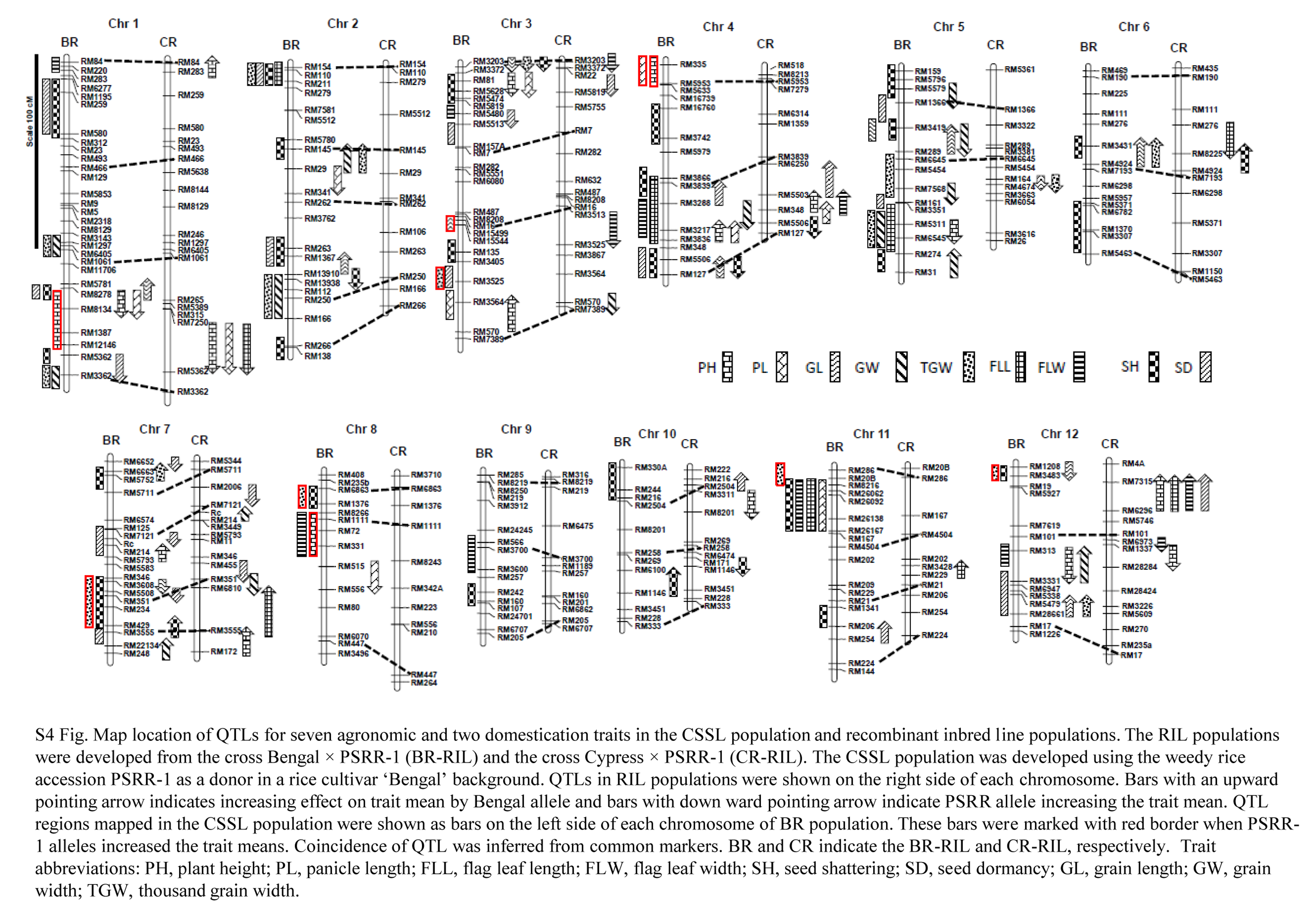

Supplement: S4 Fig — The RIL populations were developed from the cross Bengal × PSRR-1 (BR-RIL) and the cross Cypress × PSRR-1 (CR-RIL). The CSSL population was developed using the weedy rice accession PSRR-1 as a donor in a rice cultivar ‘Bengal’ background. (TIF) [file pone.0130650.s004.tif]
