# Supplementary material for: A Chromosome Segment Substitution Library of Weedy Rice for Genetic Dissection of Complex Agronomic and Domestication Traits
Source: PLoS One. 2015 Jun 18;10(6):e0130650. doi: 10.1371/journal.pone.0130650 (PMC4472838; doi:10.1371/journal.pone.0130650)
Supplement: S1 Table — (PDF) [file pone.0130650.s005.pdf]

S1 Table. Summary of the introgressed weedy rice segments and genome coverage in individual chromosome segment substitution lines developed in Bengal background.

CSSL #	No. of donor segments	No. of chromosomes with donor segments	Recurrent parental genome (%)	Homozygous segments (cM)	Heterozygous segments (cM)	Total (Homo + Hetero) (cM)	% donor genome (homozygous segments)	% donor genome (heterozygous segments)	Chromosomes bearing introgressed segments	Chr 1	Chr 2	Chr 3	Chr 4	Chr 5	Chr 6	Chr 7	Chr 8	Chr 9	Chr 10	Chr 11	Chr 12
1-1	1	1	99.39	8.75	0	8.75	0.61	0.00	1	1											
1-2	2	2	96.47	49.95	0	49.95	3.53	0.00	1, 6	1					1						
1-3	1	1	97.53	34.95	0	34.95	2.47	0.00	1	1											
1-4	1	1	98.17	35.85	0	35.85	1.83	0.00	1	1											
1-5	1	1	96.80	45.25	0	45.25	3.20	0.00	1	1											
1-6	1	1	99.02	13.85	0	13.85	0.98	0.00	1	1											
1-7	2	2	97.36	37.35	0	37.35	2.64	0.00	1, 3	1		1									
1-8	2	2	95.04	54.90	15.3	70.20	3.88	1.08	1, 5	1				1							
1-9	1	1	97.86	30.30	0	30.30	2.14	0.00	1	1											
2-1	1	1	98.32	15.45	8.35	23.80	1.09	0.59	2		1										
2-2	1	1	97.57	34.40	0	34.40	2.43	0.00	2		1										
2-3	1	1	97.25	38.85	0	38.85	2.75	0.00	2		1										
2-4	1	1	96.00	56.60	0	56.60	4.00	0.00	2		1										
2-5	1	1	98.05	27.60	0	27.60	1.95	0.00	2		1										
2-6	1	1	96.03	56.20	0	56.20	3.97	0.00	2		1										
2-7	2	2	98.61	19.65	0	19.65	1.39	0.00	2, 11		1									1	
3-1	2	2	97.26	38.75	0	38.75	2.74	0.00	3, 11			1								1	
3-2	3	3	96.90	35.55	0	35.55	3.10	0.00	3, 4, 7			1	1			1					
3-3	1	1	96.59	48.30	0	48.30	3.41	0.00	3			1									
3-4	1	1	96.70	46.65	0	46.65	3.30	0.00	3			1									

CSSL #	No. of donor segments	No. of chromosomes with donor segments	Recurrent parental genome (%)	Homozygous segments (cM)	Heterozygous segments (cM)	Total (Homo + Hetero) (cM)	% donor genome (homozygous segments)	% donor genome (heterozygous segments)	Chromosomes bearing introgressed segments	Chr 1	Chr 2	Chr 3	Chr 4	Chr 5	Chr 6	Chr 7	Chr 8	Chr 9	Chr 10	Chr 11	Chr 12
3-5	1	1	95.79	59.55	0	59.55	4.21	0.00	3			1									
3-6	2	2	94.39	79.40	0	79.40	5.61	0.00	1, 3	1		1									
3-7	1	1	97.99	28.45	0	28.45	2.01	0.00	3			1									
3-8	1	1	99.07	13.15	0	13.15	0.93	0.00	3			1									
4-1	2	2	99.04	13.55	0	13.55	0.96	0.00	4, 7				1			1					
4-2	1	1	98.78	17.25	0	17.25	1.22	0.00	4				1								
4-3	1	1	96.93	43.40	0	43.40	3.07	0.00	4				1								
4-4	1	1	96.52	49.20	0	49.20	3.48	0.00	4				1								
4-5	2	2	98.27	44.75	0	44.75	1.73	0.00	4, 6				1		1						
5-1	1	1	99.05	13.15	0	13.15	0.95	0.00	5					1							
5-2	1	1	98.30	24.05	0	24.05	1.70	0.00	5					1							
5-3	2	2	97.92	21.65	0	21.65	2.08	0.00	2, 5		1			1							
5-4	2	2	94.71	43.85	0	43.85	5.29	0.00	1, 5	1				1							
5-5	1	1	98.65	19.05	0	19.05	1.35	0.00	5					1							
5-6	2	2	96.76	45.85	0	45.85	3.24	0.00	5, 12					1							1
5-7	2	2	97.22	53.15	0	53.15	2.78	0.00	1, 5	1				1							
6-1	1	1	95.68	38.40	22.8	61.20	2.71	1.61	6						1						
6-2	1	1	96.59	41.50	6.7	48.20	2.93	0.47	6						1						
6-3	1	1	94.07	43.75	40.1	83.85	3.09	2.83	6						1						
6-4	1	1	99.07	13.20	0	13.20	0.93	0.00	6						1						
6-5	2	2	98.17	25.90	0	25.90	1.83	0.00	4, 6				1		1						
6-6	2	2	96.25	53.00	0	53.00	3.75	0.00	2, 6		1				1						
7-1	2	2	98.12	11.35	0	11.35	1.88	0.00	3, 7			1				1					

CSSL #	No. of donor segments	No. of chromosomes with donor segments	Recurrent parental genome (%)	Homozygous segments (cM)	Heterozygous segments (cM)	Total (Homo + Hetero) (cM)	% donor genome (homozygous segments)	% donor genome (heterozygous segments)	Chromosomes bearing introgressed segments	Chr 1	Chr 2	Chr 3	Chr 4	Chr 5	Chr 6	Chr 7	Chr 8	Chr 9	Chr 10	Chr 11	Chr 12
7-2	1	1	99.07	13.20	0	13.20	0.93	0.00	7							1					
7-3	2	2	96.69	35.85	0	35.85	3.31	0.00	7, 10							1			1		
7-4	1	1	98.57	25.40	0	25.40	1.43	0.00	7							1					
7-5	2	2	94.66	44.90	0	44.90	5.34	0.00	3, 7			1				1					
7-6	1	1	98.50	21.25	0	21.25	1.50	0.00	7							1					
7-7	1	1	99.60	5.65	0	5.65	0.40	0.00	7							1					
8-1	3	3	95.91	57.90	0	57.90	4.09	0.00	3, 8, 11			1					1			1	
8-2	1	1	96.28	52.80	0	52.80	3.72	0.00	8								1				
8-3	1	1	97.41	36.70	0	36.70	2.59	0.00	8								1				
8-4	1	1	96.13	54.75	0	54.75	3.87	0.00	8								1				
8-5	1	1	97.80	31.20	0	31.20	2.20	0.00	8								1				
9-1	1	1	98.52	21.00	0	21.00	1.48	0.00	9									1			
9-2	2	2	96.78	40.55	0	40.55	3.22	0.00	4, 9				1					1			
9-3	1	1	97.84	30.55	0	30.55	2.16	0.00	9									1			
9-4	1	1	98.72	18.05	0	18.05	1.28	0.00	9									1			
9-5	2	1	99.19	11.45	0	11.45	0.81	0.00	9, 9									2			
10-1	1	1	98.21	19.30	6.05	25.35	1.36	0.43	10										1		
10-2	1	1	97.16	40.25	0	40.25	2.84	0.00	10										1		
10-3	1	1	95.60	58.05	4.2	62.25	4.10	0.30	10										1		
10-4	1	1	96.89	44.05	0	44.05	3.11	0.00	10										1		
11-1	1	1	98.31	23.90	0	23.90	1.69	0.00	11											1	
11-2	2	2	94.87	72.55	0	72.55	5.13	0.00	4, 11				1							1	
11-3	2	2	95.63	61.90	0	61.90	4.37	0.00	8, 11								1			1	

CSSL #	No. of donor segments	No. of chromosomes with donor segments	Recurrent parental genome (%)	Homozygous segments (cM)	Heterozygous segments (cM)	Total (Homo + Hetero) (cM)	% donor genome (homozygous segments)	% donor genome (heterozygous segments)	Chromosomes bearing introgressed segments	Chr 1	Chr 2	Chr 3	Chr 4	Chr 5	Chr 6	Chr 7	Chr 8	Chr 9	Chr 10	Chr 11	Chr 12
11-4	1	1	97.81	24.20	0	24.20	2.19	0.00	11											1	
11-5	1	1	99.52	6.80	0	6.80	0.48	0.00	11											1	
12-1	3	3	94.70	74.95	0	74.95	5.30	0.00	2, 3, 12		1	1									1
12-2	2	2	94.35	41.05	0	41.05	4.66	0.00	1, 12	1											1
12-3	2	2	97.99	21.45	0	21.45	2.01	0.00	10, 12										1		1
12-4	2	2	97.13	33.95	0	33.95	2.87	0.00	6, 12						1						1
12-5	1	1	95.92	57.70	0	57.70	4.08	0.00	12												1
12-6	1	1	99.09	12.90	0	12.90	0.91	0.00	12												1
Total	104	103	7199.11	2619.90	103.50	2723.40	192.57	7.31	-	13	10	13	9	8	9	9	6	6	6	8	7
Mean	1.4	1.39	97.29	35.40	1.40	36.80	2.60	0.10													
