# Supplementary material for: A Chromosome Segment Substitution Library of Weedy Rice for Genetic Dissection of Complex Agronomic and Domestication Traits
Source: PLoS One. 2015 Jun 18;10(6):e0130650. doi: 10.1371/journal.pone.0130650 (PMC4472838; doi:10.1371/journal.pone.0130650)
Supplement: S2 Table — (PDF) [file pone.0130650.s006.pdf]

S2 Table. Quantitative trait loci, additive effects, and direction of phenotypic effect for plant height, panicle length, flag leaf length, flag leaf width, breaking tensile strength, germination % (arcsine transformed), grain length, grain width and thousand grain weight derived from the evaluation of the CSSLs and the recurrent parent (RP) Bengal. The presence of QTLs is inferred when there is significant difference between the means of each CSSL and the recurrent parent using the Dunnett's test. All CSSLs significantly different from the recurrent parent at p <0.01 are listed. This data was used to narrow down the QTL regions using substitution mapping. Direction of phenotypic effect (DPE) is denoted by B and R, which indicated Bengal and PSRR-1 allele increasing the trait values, respectively. In case of BTS and GermARS, reduced BTS and reduced germination % means increased seed shattering and seed dormancy.

**Plant Height (PH) (cm)**								
**CSSL #**	**Mean**	**SE**	**p-value**	**Marker interval**	**Deviation from RP**	**Additive effect**	**Additive effect contribution(%)**	**DPE**
1-8	130.5	1.53	<0.0001	RM8278-RM5362	25.4	12.7	16.7	**R**
4-1	127.2	5.54	<0.0001	RM335-RM5953	22.1	11.1	14.5	**R**
8-2	131.1	3.17	<0.0001	RM6863-RM515	26.0	13.0	17.1	**R**
**Panicle Length (PL) (cm)**								
3-6	17.4	0.78	<0.0001	RM135-RM7389	-7.8	-3.9	-5.1	B
3-7	18.7	0.37	0.0024	RM3564-RM7389	-6.5	-3.3	-4.3	B
4-1	33.9	2.47	<0.0001	RM335-RM5953	8.7	4.4	5.7	**R**
11-2	18.5	0.67	0.0014	RM286-RM167	-6.7	-3.4	-4.4	B
**Flag Leaf Length (FLL) (cm)**								
2-1	28.4	1.57	0.0076	RM154-RM211	-8.6	-4.3	-5.7	B
4-4	28.5	1.15	0.009	RM3866-RM348	-8.5	-4.3	-5.6	B
5-6	27.8	1.94	0.0028	RM3351-RM274	-9.2	-4.6	-6.0	B
11-2	25.2	1.02	<0.0001	RM286-RM167	-11.8	-5.9	-7.8	B
**Flag Leaf Width (FLW) (mm)**								
1-1	13.2	0.39	0.0071	RM84-RM6277	-2.5	-1.3	-1.6	B
3-2	12.4	0.22	<0.0001	RM5474-RM5513	-3.3	-1.7	-2.2	B
4-4	13.1	0.35	0.004	RM3866-RM348	-2.6	-1.3	-1.7	B
8-2	13.2	0.29	0.0071	RM6863-RM515	-2.5	-1.3	-1.6	B
9-2	12.5	0.70	<0.0001	RM566-RM257	-3.2	-1.6	-2.1	B
11-2	12.7	0.21	0.0003	RM286-RM167	-3.0	-1.5	-2.0	B

CSSL #	Mean	SE	p-value	Marker interval	Deviation from RP	Additive effect	Additive effect contribution(%)	DPE
12-4	12.4	0.54	<0.0001	RM7619-RM313	-3.3	-1.7	-2.2	B
**Grain Length (GL) (mm)**								
2-5	7.1464	0.05	<0.0001	RM263-RM221	-0.788	-0.394	-4.966	B
2-6	7.2608	0.11	0.0005	RM263-RM166	-0.674	-0.337	-4.245	B
3-5	8.5788	0.08	0.0012	RM5551-RM135	0.644	0.322	4.061	**R**
5-3	7.2476	0.12	0.0004	RM3489-RM289	-0.687	-0.343	-4.328	B
**Grain Width (GW)(mm)**								
1-6	2.52	0.04	<0.0001	RM1297	-0.375	-0.187	-6.474	B
1-9	2.5744	0.07	0.0017	RM5362-RM3362	-0.320	-0.160	-5.534	B
2-6	2.5452	0.02	0.0003	RM263-RM166	-0.350	-0.175	-6.038	B
5-6	2.5448	0.09	0.0003	RM3351-RM274	-0.350	-0.175	-6.045	B
**Thousand Grain Weight (TGW)(gm)**								
1-6	19.695	0.29	0.0036	RM1297	-3.119	-1.560	-6.836	B
1-9	19.525	0.51	0.0015	RM5362-RM3362	-3.289	-1.645	-7.208	B
2-1	19.353	0.58	0.0006	RM154-RM211	-3.461	-1.731	-7.585	B
2-6	18.847	0.44	<0.0001	RM263-RM166	-3.967	-1.984	-8.694	B
3-6	26.074	0.45	0.0018	RM135-RM7389	3.260	1.630	7.145	**R**
5-4	19.687	0.62	0.0035	RM289-RM3351	-3.127	-1.564	-6.853	B
5-6	19.663	0.42	0.0031	RM3351-RM274	-3.151	-1.576	-6.906	B
7-5	26.512	0.85	0.0002	RM346-RM248	3.698	1.849	8.105	**R**
8-1	26.423	0.53	0.0003	RM408-RM1376	3.609	1.805	7.910	**R**
8-2	17.909	1.04	<0.0001	RM6863-RM515	-4.905	-2.453	-10.751	B
11-1	26.219	0.45	0.0008	RM286-RM20B	3.405	1.703	7.463	**R**
12-1	27.562	0.25	<0.0001	RM1208-RM101	4.748	2.374	10.406	**R**

**Breaking Tensile Strength (BTS) (gm)**								
1-2	62.10	6.01	<0.0001	RM283-RM23	-59.3	-29.7	-39.0	B
1-7	69.05	9.37	<0.0001	RM5781-RM8278	-52.4	-26.2	-34.4	B
1-8	66.22	4.56	<0.0001	RM8278-RM5362	-55.2	-27.6	-36.3	B
1-9	67.41	3.56	<0.0001	RM5362-RM3362	-54.0	-27.0	-35.5	B

CSSL #	Mean	SE	p-value	Marker interval	Deviation from RP	Additive effect	Additive effect contribution(%)	DPE
2-1	64.32	5.29	<0.0001	RM154-RM211	-57.1	-28.5	-37.5	B
2-2	35.81	3.12	<0.0001	RM7581-RM145	-85.6	-42.8	-56.2	B
2-3	74.01	5.08	0.0007	RM5512-RM29	-47.4	-23.7	-31.1	B
2-4	53.31	4.63	<0.0001	RM145-RM3762	-68.1	-34.1	-44.7	B
2-5	48.74	7.05	<0.0001	RM263-RM221	-72.7	-36.3	-47.7	B
2-6	62.64	2.34	<0.0001	RM263-RM166	-58.8	-29.4	-38.6	B
2-7	70.99	5.49	0.0002	RM266-RM138	-50.4	-25.2	-33.1	B
3-1	38.23	3.44	<0.0001	RM3203-RM5819	-83.2	-41.6	-54.7	B
3-2	78.70	7.17	0.0041	RM5474-RM5513	-42.7	-21.4	-28.1	B
3-5	77.31	5.55	0.0035	RM5551-RM135	-44.1	-22.0	-29.0	B
3-6	65.40	7.27	<0.0001	RM135-RM7389	-56.0	-28.0	-36.8	B
4-2	73.28	9.13	0.0005	RM3742	-48.1	-24.1	-31.6	B
4-3	65.82	6.59	<0.0001	RM3742-RM3839	-55.6	-27.8	-36.5	B
4-4	67.89	5.76	<0.0001	RM3866-RM348	-53.5	-26.8	-35.2	B
4-5	29.28	3.04	<0.0001	RM348-RM127	-92.1	-46.1	-60.5	B
5-1	68.02	5.16	<0.0001	RM159-RM5579	-53.4	-26.7	-35.1	B
5-2	70.84	4.23	0.0002	RM159-RM1366	-50.6	-25.3	-33.2	B
5-3	74.58	6.20	0.0016	RM3419-RM289	-46.8	-23.4	-30.8	B
5-6	56.68	4.29	<0.0001	RM3351-RM274	-64.7	-32.4	-42.5	B
5-7	79.96	5.39	0.0064	RM274-RM31	-41.5	-20.7	-27.2	B
6-2	56.24	4.28	<0.0001	RM225-RM4924	-65.2	-32.6	-42.8	B
6-3	61.74	3.14	<0.0001	RM111-RM7193	-59.7	-29.8	-39.2	B
6-6	67.08	4.28	<0.0001	RM5957-RM5463	-54.3	-27.2	-35.7	B
7-1	61.04	3.77	<0.0001	RM6652-RM6663	-60.4	-30.2	-39.7	B
7-2	53.65	3.62	<0.0001	RM5711	-67.8	-33.9	-44.5	B
7-5	79.30	7.72	0.0051	RM346-RM248	-42.1	-21.1	-27.7	B
8-1	50.31	3.96	<0.0001	RM408-RM6863	-71.1	-35.6	-46.7	B
8-2	68.81	6.91	<0.0001	RM6863-RM515	-52.6	-26.3	-34.6	B
9-3	68.09	10.59	<0.0001	RM257-RM107	-53.3	-26.7	-35.0	B
10-1	67.88	4.73	<0.0001	RM330A-RM216	-53.5	-26.8	-35.2	B
10-2	73.54	7.47	0.0006	RM330A-RM8201	-47.9	-23.9	-31.5	B
10-3	66.07	6.17	<0.0001	RM330A-RM269	-55.3	-27.7	-36.4	B
11-2	42.28	3.71	<0.0001	RM286-RM167	-79.1	-39.6	-52.0	B

CSSL #	Mean	SE	p-value	Marker interval	Deviation from RP	Additive effect	Additive effect contribution(%)	DPE
11-4	77.31	9.88	0.0025	RM1341-RM254	-44.1	-22.1	-29.0	B
12-1	68.74	5.66	<0.0001	RM1208-RM101	-52.7	-26.3	-34.6	B
**Germination % (GermARS)**								
1-2	58.90	4.49	0.0007	RM283-RM23	-24.5	-12.2	-16.1	B
1-7	51.33	6.02	<0.0001	RM5781-RM8278	-32.0	-16.0	-21.0	B
1-8	62.40	5.34	0.0089	RM8278-RM5362	-21.0	-10.5	-13.8	B
2-1	58.71	1.95	0.0006	RM154-RM211	-24.7	-12.3	-16.2	B
3-2	53.19	5.25	<0.0001	RM5474-RM5513	-30.2	-15.1	-19.8	B
3-3	56.09	3.88	0.0003	RM5513-RM6080	-27.3	-13.6	-17.9	B
3-6	24.67	3.58	<0.0001	RM135-RM7389	-58.7	-29.4	-38.6	B
4-5	45.50	4.45	<0.0001	RM348-RM127	-37.9	-18.9	-24.9	B
5-2	59.54	7.70	0.0012	RM159-RM1366	-23.8	-11.9	-15.7	B
5-5	52.04	3.14	<0.0001	RM161-RM3351	-31.3	-15.7	-20.6	B
5-6	37.08	9.20	<0.0001	RM3351-RM274	-46.3	-23.1	-30.4	B
7-3	49.68	3.51	<0.0001	RM6574-RM5583	-33.7	-16.8	-22.1	B
7-4	40.37	1.59	<0.0001	RM125-RM5793	-43.0	-21.5	-28.3	B
7-5	36.43	2.37	<0.0001	RM346-RM248	-46.9	-23.5	-30.8	B
7-6	61.15	3.74	0.0039	RM3555-RM248	-22.2	-11.1	-14.6	B
12-5	45.17	6.41	<0.0001	RM7619-RM6947	-38.2	-19.1	-25.1	B
