# Supplementary material for: A Chromosome Segment Substitution Library of Weedy Rice for Genetic Dissection of Complex Agronomic and Domestication Traits
Source: PLoS One. 2015 Jun 18;10(6):e0130650. doi: 10.1371/journal.pone.0130650 (PMC4472838; doi:10.1371/journal.pone.0130650)
Supplement: S3 Table — (PDF) [file pone.0130650.s007.pdf]

S3 Table. Phenotypic correlation coefficients (Pearson coefficient) among the traits in BR CSSL population (n=74) and RIL populations. The top right half of the table represents values calculated for the CR-RIL (normal font) and BRRIL (bold font) populations. Correlation coefficients calculated for the CSSL population is given in the bottom half. *, **, *** indicates significance at P <0.001, P <0.01, and P <0.05, respectively. NA: data not available.

Trait	PH	PL	FLL	FLW	SH or BTS[a]	GermARS	GL	GW	TGW
PH		0.753***<br>**0.583*** **	0.679***<br>**NA**	-0.045<br>**NA**	-0.251**<br>**-0.102**	0.037<br>**-0.038**	0.073<br>**-0.074**	0.019<br>**-0.112**	0.107<br>**-0.028**
PL	0.540***		0.723***<br>**NA**	0.149<br>**NA**	-0.176<br>**-0.026**	-0.022<br>**-0.081**	0.158<br>**0.067**	-0.055<br>**-0.166* **	-0.108<br>**-0.025**
FLL	0.592***	0.507***		0.061<br>**NA**	-0.217*<br>**NA**	-0.024<br>**NA**	0.153*<br>**NA**	0.019<br>**NA**	0.175*<br>**NA**
FLW	0.228	0.031	0.452***		-0.012<br>**NA**	-0.249**<br>**NA**	0.022<br>**NA**	0.087<br>**NA**	0.164*<br>**NA**
SH or BTS	-0.033	-0.009	0.143	0.002		-0.018<br>**0.072**	-0.224**<br>**-0.154**	-0.090<br>**0.042**	-0.232**<br>**-0.022**
GermARS	0.144	-0.104	0.149	0.110	0.157		0.148*<br>-0.043	-0.145<br>**0.001**	0.093<br>**-0.012**
GL	0.022	0.226	0.329**	0.101	0.183	-0.004		-0.132<br>**0.071**	0.555***<br>**0.457*** **
GW	-0.117	0.128	0.151	-0.081	-0.007	0.150	0.569***		0.401***<br>**0.537*** **
TGW	-0.149	-0.003	0.144	-0.022	0.145	-0.011	0.736***	0.679***	

PH, plant height; PL, panicle length; FLL, flag leaf length; FLW, flag leaf width; SH, seed shattering; BTS, breaking tensile strength; germARS, arcsine transformed value of germination%; GL, grain length; GW, grain width; TGW, thousand grain width.

[a] Seed shattering scores were used in RIL populations but BTS values were used in CSSL population.
